# Traditional Dietary Knowledge of a Marginal Hill Community in the Central Himalaya: Implications for Food, Nutrition, and Medicinal Security

**DOI:** 10.3389/fphar.2021.789360

**Published:** 2022-03-30

**Authors:** S. N. Ojha, Aryan Anand, R. C. Sundriyal, Deepshikha Arya

**Affiliations:** ^1^ G.B. Pant National Institute of Himalayan Environment, Kosi-Katarmal, Almora, India; ^2^ Department of Forestry and Natural Resources, HNB Garhwal University, Srinagar, India; ^3^ Regional Ayurveda Research Institute, Ranikhet, India

**Keywords:** central Himalaya, traditional food crops, dietary intake, food–medicine interface, nutritional security, health care, traditional cultural knowledge, Uttarakhand

## Abstract

Himalayan communities illustrate a rich agriculture–medicine use system that not only provides adequate dietary diversity and nutrition but also delivers therapeutic security. This study explores the food–medicine interface as observed by the marginal hill communities in the central Himalaya with an aim to assess traditional agriculture and food plants with relation to dietary diversity and nutritional and medicinal values based on comprehensive research. A total of 445 respondents were interviewed to obtain data on food intakes using dietary recall methods and dietary diversity indices (DDIs). The ethnomedical use of plant species was gathered from respondents as well as from various published studies for respective species. Nutritional parameters were collected from the Indian Food Composition Table developed by the ICMR, India to analyze the average nutritional intake. The traditional food system achieves the dietary and nutritional needs of the community within the standard norms. The average household dietary diversity of 7.45, 7.34, and 8.39 in summer, monsoon, and winter seasons, respectively, sustain 79, 74, and 93% of energy requirements in respective, seasons. The average food consumption score (FCS) was 73.46, and all the food exhibited rich phytochemicals, such as amino acids, alkaloids, carotenoids, flavonoids, glycosides, and phenolic acids. These plants also provided effective treatments against several ailments and illnesses, such as cardiovascular diseases, diabetics, gastrointestinal issues, and inflammation The indigenous cuisines also have significant food and medicinal values. Considering that the community had significant knowledge of food systems with their nutritional and therapeutic utility, there is a need to protect and document this indigenous knowledge. Also, most of the crops are still under cultivation, so there is a need to create more awareness about the nutritional and therapeutic value of the system so that it could be retained intact and continued. The implications of this research are of both academic importance and practical significance to ensure food–medicine security and avoid malnutrition among rural communities. It is expected that the study would lead to renewed thinking and policy attention on traditional agriculture for its role in food and nutritional security that may lead to a sustainable food supply system.

## Introduction

Notwithstanding significant growth in the agriculture sector in the past few decades, still, there is persistent hunger and malnutrition in many parts of the world (FAO 2017). Expanding the food production system to new areas comes with a heavy natural environmental cost, thus posing a challenge to sustainable food supply to the ever-growing population (Fróna et al., 2019). It is disheartening to note that despite the global commitment to bring food security and end hunger and malnutrition by 2030, the world is far from achieving these SDG objectives; on the contrary, the number of undernourished and hungry people has been increasing (FAO, IFAD, UNICEF, WFP and WHO, 2020). There is also a decline in the access of quantity and quality of food in many places. Disruptions in food supply and income greatly impact the access of poor and vulnerable people to nutritious foods and healthy food across the globe (FAO, IFAD, UNICEF, WFP and WHO, 2018). As per the FAO report, nearly 11% of the global population and 14.5% of India’s population is undernourished. An Indian Council of Medical Research (ICMR) report emphasized that malnutrition is a major contributing factor behind the death of children below 5 years of age, and the incidences of malnutrition are high in rural and tribal areas (Narayan et al., 2019). The major cause of malnutrition is lack of adequacy of fresh fruits, vegetables, legumes, grains, meat, and milk. Fortunately, there are many traditional crops and food production systems that are in place for centuries and have been meeting food and nutritional requirement of a substantial population (Adhikari et al., 2019). Such agricultural systems are well-established and sustainable in food supply; thus, they can provide competitive benefits over the modern agriculture systems (Carl et al., 2017). All over the world, traditional food systems are supportive in maintaining local food habits and culture along with conserving vital food and fodder plants and their wild relatives (FAO 2011a; Khoury et al., 2016). It is largely practiced by smallholders, particularly by women, and is important in sustaining agricultural genetic diversity and soil fertility (FAO 2011b; Maikhuri et al., 2015). Other important aspects of traditional agriculture are withstanding nutritional and food security, ensuring optimum productivity and economic return, reducing the vulnerability of crops during adverse conditions, retaining natural resource base, and minimizing adverse environmental impacts (Sundriyal et al., 1994; ICMR-ICFT 2017). Traditional food systems are considered beneficial in terms of providing fewer calories and saturated fat, more iron, calcium, zinc, and vitamin A (IFPRI 2015). It also provides relief in selected health issues, such as allergies, asthma, digestive and cardiovascular illness, obesity, and even diabetes, thus acting as an interface of food and medicine (CINE 2021). The traditional crops and wild plants offer wide food diversity and nutritional security to local communities, thus safeguarding against hunger (Jones 2017). It secures access of marginal communities to adequate food especially in the low-income regions where a significant share of the population, especially women, is still engaged in agriculture (Bisht et al., 2018). Local food systems also offer enriched dietary diversity and quality that strengthen environmental sustainability (Fanzo et al., 2013) and therapeutic efficacy (Sarkar et al., 2015). Thus the traditional food and dietary systems constitute a backbone for the sustainable development of agriculture, food security, and poverty alleviation. Therefore, supporting the diversity of foods and species consumed in local diets has significant benefits for sustainable food systems and public health perspectives (Jennings et al., 2015; Hettiarachchi 2021). However, globally, there is a net decline in traditional food systems despite the fact that a significant population still depends on them (Khoury et al., 2014). The developing countries are undergoing a rapid change in land uses and governance with a shift from subsistence to commercial agriculture to accelerate economic growth that ultimately affects the access of local communities to food (Broegaard et al., 2017). A decline in crop diversity has significant worldwide implications for food and nutritional security. This increases the demand to make traditional agriculture more reliable by generating greater awareness regarding such systems that not only meet food demand but also serve as a better example of an interface of food and medicine. The present study was undertaken with a similar focus so as to create an enhanced understanding of the advantages and limitations of traditional agriculture in the central Himalayan region. Here, a large section of the population is still dependent on traditional agriculture, and women have been a major workforce to perform agricultural activities (Sundriyal et al., 2014). We argue that there is a need to highlight the health, nutritional, and therapeutic benefits of the traditional food system to revive it from disappearance. Considering this, the present study investigates the food–nutrition–medicine interface of marginal hill communities in the central Himalaya. The objectives are to assess 1) crops and wild plant diversity used to fulfill the dietary quality of the Central Himalayan community and 2) nutrient and therapeutic claims of the traditional diets essential for sustenance of the community. We expect that a better understanding of the intersection between traditional food, dietary diversity, nutritional quality, and medicinal efficacy along with some possible developmental options, thus, would receive greater attention from policy planners and developmental workers. In addition to this, it may salvage the dwindling traditional dietary system that is further leading the marginalization of the smallholder farming communities.

### Study Region and Methods

**FIGURE 1:**
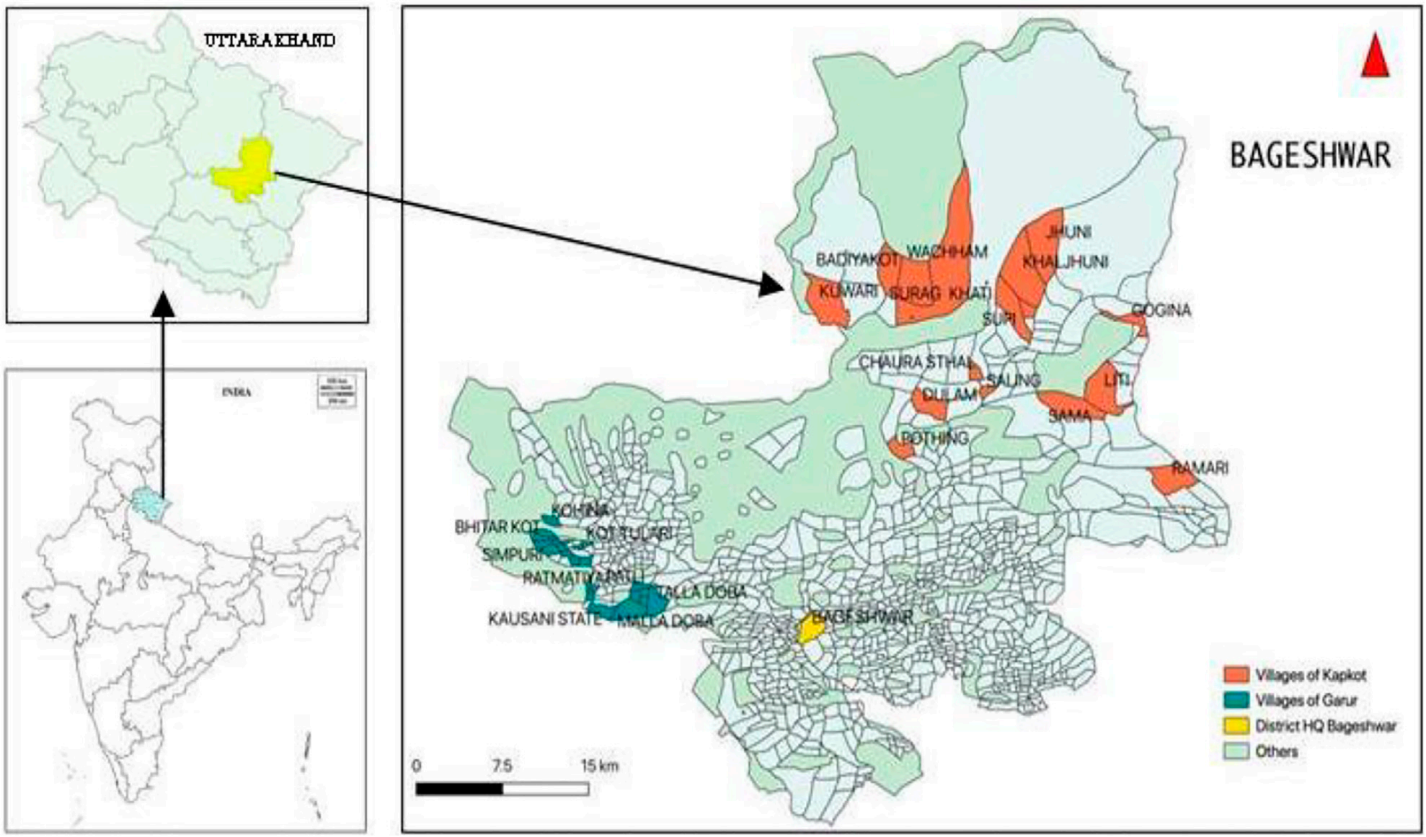
Study area and villages in Garur and Kapkot Blocks of Bageshwar, Uttarakhand, India.

For this study, the Uttarakhand state in the central Himalaya, India was targeted that comprised a total geographical area (TGA) of 53,483 km^2^. The state shares borders with Himachal Pradesh in northwest & Uttar Pradesh in the south and international borders with Tibet in the north and Nepal in the west. Physiographically, Uttarakhand can be divided into three broad zones, viz., the Himalayas, the Siwalik, and the Terai region. Administratively, the state has 13 districts and houses a population of 10.09 million (69.77% rural and 30.23% urban) with a livestock population of 4.79 million and largely exhibits an agrarian and pastoralist economy with high dependence on forest resources. For detailed investigation, we selected the Bageshwar district (area of 2,302 km^2^) of Uttarakhand that is most centrally located and represents all broad features of the state (Figure 1). Bageshwar is largely hilly terrain covering Siwalik ranges and the high Himalayas. Pindar, Saryu (Sarju), Gomati, and Pungar are the main rivers flowing across the district. Administratively, the district comprised four Tehsils, viz., Bageshwar, Kapkot, Kanda, and Garur and three blocks, viz., Bageshwar, Garur, and Kapkot. As per the 2011 census, the total population is 259,898 (male 48% and female 52%) with 96% living in the rural areas. There are 874 inhabited villages in the district (Anonymous 2011). The community of the area is divided into three categories, viz., General, Scheduled Class, and Scheduled Tribe. The education status of the district is good, with an overall literacy rate of 80.01%, although it is much higher for males (92.33%) than females (69.03%). The district has 10.8% land area under agriculture, which is mainly rain-fed (average annual rainfall of 1,200–1,400 mm). Only 20% of agricultural land is irrigated. In addition, the district comprised 55% land area under forest, 5.42% cultivable barren land, 3.23% barren and uncultivable land, and 10.64% as permanent pasture and grazing land (Anonymous 2011). The district also has 273,051 livestock that forms an integral part of each household. These animals comprised cattle (37%), buffalo (10%), goats (40%), sheep (6.5%), and others (6.5%). In addition, there is also backyard poultry. As per the livestock census 2012, Uttarakhand exhibited low per capita availability of milk (387g/day/person), meat (2kg/person/year), and egg (27eggs/person/year); therefore, in rural places, there is high dependence on agriculture. However, the economy is collectively met from all these lands and largely subsistence-type. The majority of people are involved in the primary sector (agricultural activities), although some also work in secondary and tertiary sectors, such as private works, businesses, and government jobs. Generally, the community is greatly dependent on farming and natural resources and characterized as highly marginal with small and scattered land holdings, small production, and low income. The major foods of the community are rice, finger millet, wheat, barley, maize, pulses, and a wide variety of vegetables cultivated or collected from the wild. Occasionally, people also consume animal products (meat, ghee, buttermilk, milk, curd, etc.). Generally, the male population out migrates to earn better livelihoods, leading to dominance of womenfolk in the villages. It also results in a continuous increase in fallow lands and culturable wastelands. The district has limited health infrastructure mainly located in urban areas. As per the National Family Health Survey (2015–16), the prevalence of malnutrition in Uttarakhand among children under 5 years of age was 26.6% underweight and 33.5% stunting (Anonymous 2014). As per Food Policy Research data, the status of nutrition in Bageswar revealed that among the children <5 years, 23% exhibited stranded growth, 26.3% were underweight, and 45% anemic. In women of reproductive age, 41.3% were anemic while 24.9% had a body mass index <18.5 kg/m^2^. For common health needs, rural communities are largely dependent on the traditional health care system (Ojha et al., 2020).

### Assessing Traditional Food and Medicinal Usage

The study was conducted from 2017 to 2019. To collect field data, we randomly selected 24 villages covering Garur Ganga, Saryu, and Gomati Valley of Garur and Kapkot blocks of the district. The selection of villages covered an altitude from 1,200 to 2,700 m above sea level. Largely, the area has a similar sociocultural structure though crops and plants vary with elevation. The criterion for village selection was to maximize the probability of including respondents from different altitudes, social statuses, and castes. Owing to the topography of the region, the villages lie on the fringes of the hills as well as in the high peaks of the region. Thus, it was assured that a probabilistic approach was to be taken. A detailed questionnaire was prepared to cover various broad areas of food intake. In addition, focused interviews were also arranged regarding knowledge, attitude and practices, dietary intake, and food habits. Since most farmers owned small landholding and were categorized as smallholders, for selecting a household (participant), we initially conducted a household listing operation in each selected village to provide a frame to include in the sample. Accordingly, a total of 25% households or a maximum of 30 households were sampled in each village with equal probability. Altogether, we sampled a total of 445 households across villages. Of the total respondents, 65% were women being the major workforce as men migrated to towns and cities for finding alternate employment. The median age of the interviewees was 38. A detailed list of all traditional agricultural practices, crops, and foods eaten by the local community, the local name of the species, its source of availability (cultivated or collected from wild), plant part used, the month of collection, seasonal consumption pattern, uses other than food during illness, and mode of use and application was prepared. We also explored major crops and cropping patterns in rain-fed and irrigated fields. As the majority of crop species were common, they were simply matched with the existing herbarium for identification purposes. We used standard plant nomenclature using IPNI (2021). An important part of the study was to assess the food and medicine interface of traditional crops and plants that are most commonly used by the marginal hill community. These practices are being used since eternity, descended from the inherited knowledge of the locals and indigenous population of Uttarakhand.

As women commence most household doings and take key decisions in food making, of the total respondents, a total of 100 women were targeted for 24-h dietary recall survey (FAO, 2018). These women were of the age group of 25–50 years. The study was repeated consecutively for three major seasons, viz., summer (March to June), monsoon (July to October), and winter (November to February). Common village measuring units (such as *patha*) were standardized to obtain an actual quantity of food intake by the participants to estimate the usual daily intake of food, and the frequency of intake was combined to obtain the optimum digits. Information on main ingredients and cooking methods was gathered for different traditional food recipes, cuisines, or herbal preparations. We considered those dishes that have been traditional in nature and were in practice through generations. The frequency of food intake for different species is gathered in three categories: daily (consumed regularly), weekly (consumed at least once in a week), and occasional (consumed at least once in a month or season). We considered median values of intake instead of mean to avoid skewness of data. As some species are preferred for healthcare also, we also gathered information on the illness being treated, therapy applied, and techniques used in treatment. To further assess the potency of a treatment using local crops and plants, we also interviewed local *Vaidyas* (herbal healers or folk medicine practitioners) using snowball sampling. It was through discussion with locals and other practitioners that we identified 30 healers who agreed to share information. Later on, they were interviewed to elucidate the potency of crops/plants for therapeutic purposes. For this, two general meetings (one in each valley) were organized in which the information gathered from the community was shared with vaidyas to provide a common viewpoint on the effectiveness of the dietary habit for treatment of illness/ailments. In addition, the healers also have knowledge and experience on the use of different crops and wild plants for its use in curing diseases and ailments. Other than vaidyas, 20 members of the native community also participated in each meeting. Information was gathered on the illness being tackled, plant parts used, processing (if any), and mode of application. All the data collected as above were arranged by listing all crops and wild plants used as food, dietary diversity and food groups consumed in different seasons, preference of traditional food in terms of nutrition and medicinal utility, and sociocultural significance of food items, if any.

### Assessing Dietary Diversity Indices

Dietary diversity indices (DDI) provide us with quantified variables with outcomes that define the quality of diet taken by the communities. The study used two major indices in the study to quantify the quality and diversity of food taken by the community. The household dietary diversity score (HDDS) was used to check the food consumption and household access to a variety of food as well as a proxy for nutrient adequacy. Data were collected in accordance with the guidelines for measuring household and individual dietary diversity and with suggested 12-food group indicators (FAO, 2010). For all the three seasons (summer, monsoon, and winter), a 24-h dietary recall was taken from the respondents which was converted into Boolean variables (where 0 = food not taken and 1 = food taken) and finally summing up the values to obtain the HDDS. Another index implemented in the study is the Food Consumption Score (FCS) developed by the World Food Program to aggregate household-level data on diversity and frequency of food consumed during a specific duration (WFP, 2009). The indicator used a 7-day dietary recall and frequency of the food groups whose intake frequencies were noted in the questionnaire and after applying the respective weights to the food groups.

FCS was calculated using Equation 1 as follows:

 FCS=A_staple X_staple+A_pulses X_pulses+A_veg X_veg+A_fruits X_fruits+A_meat X_meat+A_sugar X_sugar+A_dairy X_dairy+A_oils X_oils, (1)

where,

a¬i=Weight of each group (Starch staples=2, Pulses=3, Vegetables=1, Fruits=1, Meat/fish/eggs=4, Milk=4, Sugar/Sweets=0.5, Fat=0.5).

xi = frequency of food consumed in past 7 days.

The nutrient adequacy ratio (NAR) was also calculated for the 13 nutritional indicators (viz., energy, proteins, carbohydrates, fats, vitamin B1, vitamin C, vitamin D, vitamin E, Ca, Mg, Fe, Na, and Zn) used in the study by dividing the participant’s actual intake of each nutrient by the recommended dietary allowance (RDA), and additionally the mean adequacy ratio (MAR) was calculated using the following equation (INDEX Project, 2018):

MAR=ΣNAR/(Number of Nutrients)

### Assessing Nutritional Status of Food and Medicinal Plants

Food plants comprise significant nutritional and health benefits as they contain all the essential nutrients in the leaves, roots, stems, flowers, and fruits of many plants. Therefore, after collecting the information on traditional food plants and food habits of the communities, selected crops were assessed for their nutritional values from the Indian food composition developed by the ICMR, India (ICMR-ICFT 2017; Longvah et al., 2017). The species-specific values for energy, proteins, fats and carbohydrates, water-soluble and insoluble vitamins (such as thiamine—vit. B1; ascorbic acid—vit. C; ergocalciferol—vit. D; and tocopherol—vit E), and minerals (such as Ca, Fe, Mg, Na, and Zn) were undertaken. For some species, nutritional parameters were assessed from other research sources derived by searching Google Scholar and PubMed sources. The intakes, as classified on the basis of daily, weekly, and occasionally, were filtered and analyzed with respect to the nutritional information to obtain the average nutritional intake of individuals, although the rarely eaten foods were not included in the consumption analysis.

## Results

### Richness of the Food System

**FIGURE 2:**
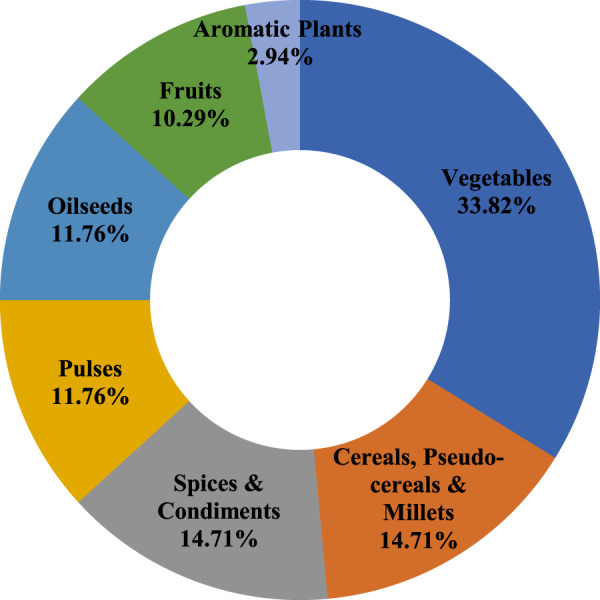
Categories of agri-horticultural plant species used in food, nutrition, and primary health care.

It was recorded that the community used as many as 68 food plant species comprising cereals, pseudo-cereals, millets, vegetables, pulses, fruits, spices and condiments, oilseeds, and medicinal and aromatic plants to fulfill their basic needs (Table 1). The main crops were rice (comprise 50% of agricultural land), finger millet (20%), wheat (19%), barley (5%), and maize (1.35%), and the cropping pattern differs in irrigated and unirrigated (rain-fed) agricultural lands. In irrigated fields, rice forms the major crop, although in some areas, wheat is also grown. In unirrigated fields, however, mixed cropping along with crop rotation has been the key feature. In such areas either dry rice, followed by wheat and millet, or millet followed by rice and wheat cultivation is followed on a 3-years crop rotation basis. Mix-cropping of pulses (lentil, urd, etc.) and oilseeds (mustard, sesame, soybean, etc.) was integral in rain-fed lands. Besides, the cultivation of a wide variety of minor crops, vegetables, and medicinal plants was also performed by the farmers. The main fruits of the study area were citrus, pear, mango, walnut, apple, peach, plum, apricot, litchi, etc. Most species were cultivated as vegetables (33.82%), followed by cereals and millets (15%), spices & condiments (14.71%), oilseeds (11.76%), fruits (10.29%), and aromatic species (2.9%) (Figure 2). A total of 35 species were also sold in local markets. In addition, a significant number of wild plants were collected from nearby forests to fulfill diverse needs.

**TABLE 1:** Crops plants used as traditional foods, nutritional security, and primary healthcare by central Himalayan communities.

	Crop category and scientific name {family, (RKT no.#)}	Common name		Local name	^$^Availability season	Cultivated or collected from the wild	Additional use other than food (during illness)	Mode of use or application	Details of recipes or medicinal use
A	Cereals, pseudo-cereals and millets								
1	*Amaranthus caudatus* L. {Amaranthaceae (RKT 25885)}		Amaranth/Ramdana	*Kedari chuwa*	S, W	C	Measles	DA	Seeds (25 g) are spread over the sleeping bed
2	*Echinochloa frumentacea* Link. {Poaceae (RKT 7475)}		Barnyard millet	*Jhangora/Madira*	S, W	C	Anemia	Co	De-husked seeds and flour used as *chapati* (bread) and cooked rice, respectively
3	*Eleusine coracana* (L.) Gaertn. {Poaceae (RKT 7299)}		Finger millet	*Madua*	M, W	C	Cold and cough and high blood pressure	Co	Porridge of flour and breads (hot *chapatti* 2–3 nos. for 3–4 days)
4	*Fagopyrum esculentum* Moench {Polygonaceae (RKT 27688)}		Buckwheat/Kuttu	Ogal/phaphar	S, W	C	Energy booster	Co	Recipes (halwa, chapati, and vegetables) eaten for boosts of energy
5	*Fagopyrum cymosum* (Trev.) Meisn {Polygonaceae (RKT 12896)}		Wild buckwheat	Jhankara	S	W	Stomachic	Co	Leaves and tender twigs used as vegetable
6	*Hordeum vulgare* L. {Poaceae (RKT 7855)}		Barley	*Jau*	S, M	C	High blood pressure and throat disorders	S, Co	Breads (*Chapatis*-25 g/person) for blood pressure normalization; smoke of burning grains (10 g) inhaled for throat cure
7	*^a^* *Oryza sativa* L. {Poaceae (RKT 4796)}		Paddy	*Dhan*	S, M, W	C	Leucorrhea	Co	Boiled rice of *Sanwdhan* (100–200 g/person) locally known as *Bhaat*
8	*Setaria italica* (L.) P. Beauv. {Poaceae (RKT 7389)}		Foxtail millet	*Kauni*	S, M, W	C	Measles	Co	De-husked grains (50–100 g/individual) are cooked as rice and served to the patients
9	^a^ *Triticum aestivum* L. {Poaceae (RKT 26973)}		Wheat	*Gehun*	S, M, W	C	--	Co	Seed flour used as chapatti and other traditional dishes
10	*Zea mays* L. {Poaceae (RKT 7536)}		Maize	*Makka*	S, M	C	Whooping cough	AF	Blank cob’s ash (20–30 g)
B	Vegetables								
11	*Asparagus filicinus* Buch.-Ham. ex D.Don {Asparagaceae (RKT 14469)}		*Asparagus*	*Kairuwa*	S	W	Energy booster and tonic	Co	Young shoots (40–100 g/individual) vegetable
12	*Bauhinia variegata* L. {Fabaceae (--)}		Kachnar	*Kwairal*	S	W	Dysentery, diarrhea, and Stomachic	Co	Boiled flower buds used as a traditional *Rayata* and pickle
13	*Benincasa hispida* (Thunb.) Cogn. {Cucurbitaceae (RKT 26003)}		Wax gourd	*Paitha/Kumila/Bhuj*	S, M	C	Stomachic	Co	Fruit used in traditional *Baris* and sweet dishes
14	*Brassica oleracea* var. capitata L. {Brassicaceae (--)}		Cabbage	*Band gobi*	W	C	--	Co	Vegetative bud used as vegetable
15	*Chenopodium album* L. {Amaranthaceae (RKT 19173)}		*Chenopodium*	*Bathua*	W	C	Cold and cough	Co	Soup of (100 ml) of matured grains (25 g) with normal spices
16	*Colocasia esculenta* (L.) Schott {Araceae (RKT 8706)}		Taro	*Gaderi/Pinalu*	M, W	C	--	Co	Corms, rolled leaf blade, and petiole or leaf stalk used as vegetable
17	*Cucumis sativus* L. {Cucurbitaceae (RKT 1040)}		Cucumber	*Kakari*	S, M	C	Sun stroke and malaria	In	Water of matured cucumber fruits
18	*Cucurbita moschata* Duch. ex Poir. {Cucurbitaceae (--)}		Pumpkin	*Kaddu*	M,W	C	--	Co	Green and matured (ripe) fruit vegetable (60–100 g/individual)
19	*Cyclanthera pedata* (L.) Schrad {Cucurbitaceae (RKT 27159)}		Wild cucumber	*Meetha/RamKarela*	S, M	C	Liver diseases and stomachic	Co	Fruit (100 g/individual) vegetable
20	*Diplazium* esculentum (Retz.) Sw. {Athyriaceae (--)}		Fern	*Lingura*	S, M	W	Constipation	Co	Young fronds (50 g/individual) used as vegetable
21	*Dioscorea alata* L. () {Dioscoreaceae (RKT 11878)}		Winged yam	*Tarur/Tairu*	W	W	Stomachic	Co	Tuber used in traditional recipe—*Tarur ki sabzi* (50 g/individual)
22	*Dioscorea bulbifera* L. {Dioscoreaceae (RKT 27263)}		Dioscorea, Yam	*Genthi*	W	C	Cold and cough	Co	Cooked vegetables of yams (150 g/individual)
23	*Lagenaria siceraria* (Molina) Standl. {Cucurbitaceae (--)}		Bottle gourd	*Lauki*	S, M	C	Low and high blood pressure	Co	Juice or soup (85–105 g/individual) of vegetable
24	*Luffa acutangula* (L.) Roxb. {Cucurbitaceae (RKT 3602)}		Ridge gourd	*Torai*	S, M	C	Fever	Co	Fruit (85–105g/individual) vegetable
25	*Megacarpaea polyandra* Benth. {Brassicaceae (RKT 1378)}		Rooki	*Barmola/Rookhi/Rugi*	S	W	Dysentery, fever, and stomach ache	Co, DA	Fresh leaves (70 g/individual) used as vegetable; roots eaten raw
26	*Momordica charantia* L. {Cucurbitaceae (RKT 24932, RKT 27529)}		Bitter gourd	*Karela*	S,M	C	Stomach ache, diabetes, and antiparasitic	Co, In	Fresh fruit juice (10–20 ml/day for 3–4 days in a week; Fruit (50 g/individual) vegetable
27	*Phytolacca acinosa* Roxb. {Phytolaccaceae (--)}		Indian pokeweed	*Jarag*	S, M	C	Cough, cold, and constipation	Co	The fresh tender leaves and twigs (40–50 g/individual) are cooked as vegetable
28	*Raphanus sativus L.* (Brassicaceae) {Brassicaceae (RKT 10925, RKT 27049)}		Radish	*Mooli*	M, W	C	Jaundice	Co	Green leaves and roots (50–70 g/individual) cooked as vegetables without oil and turmeric
29	*Solanum melongena* L. {Solanaceae (RKT 29242)}		Brinjal	*Baigan*	S, M	C	Dog bite/rabies	AF	Stem wood ash (20–30 g) powder is tied on the wounds of biting spot
30	*^a^* *Solanum tuberosum* L. {Solanaceae (RKT 8138)}		Potato	*Aalu*	S, M, W	C	--	Co	Starchy food (king of the vegetable), ingredients of all type vegetable
31	*Spinacia oleracea* L. {Amaranthaceae (RKT 28871)}		Spinach	*Palak*	W	C	Hemoregulatory	Co	Leaf used as traditional dish—*Palak ka Kafa*
32	*Trichosanthes anguina* L. {Cucurbitaceae (RKT 2264)}		Snake gourd	*Chichanda*	S, M	C	Fever	Co	Vegetables of fruits (70 g/person)
33	*Urtica ardens* Link. {Urticaceae (RKT 24064)}		Himalayan nettle	*Bichhughas Kandali/*	S, M, W	W	Menorrhagia disorder and muscular pain	Co, DA	Young shoots are cooked as vegetable and eaten for smooth menstruation; young shoots applied in body cramp & external pains (muscular pain)
**C**	Pulses								
34	*Glycine max* (L.) Merrill (Fabaceae) {Fabaceae (RKT 29313)}		Soybean	*Bhatt*	S, M, W	C	Jaundice and piles	Co	Local recipes like *Jaula, Dubuke*, and *Chutkani* are prepared from the grains (40 g/individual)
35	*Glycine max subsp*. soja (Sieb. & Zucc.) H. Ohashi {Fabaceae (RKT 15664)}		Black soybean	*Kala Bhatt*	S, M, W	C	Jaundice and piles	Co	Local recipes like *Jaula, Dubuke*, and *Chutkani* are prepared from the grains (45 g/individual)
36	*Lens culinaris Medik* {Fabaceae (RKT 7781)}		Lentil	*Masoor*	S, M, W	C	Anemia	Co	Local recipes like *Dal and Dubuke* are prepared from the grains (40 g/persons)
37	*Phaseolus vulgaris* L. {Fabaceae (RKT 29059)}		French Bean	*Rajma*	S, M, W	C	Energy booster (protein rich)	Co	Local landraces are good sources of protein used as different recipes
38	*Vigna mungo* (L.) Hepper {Fabaceae (RKT 27199)}		Black gram/urad	*Maas*	S, M, W	C	Energy booster and for fracture	Co, In	Local recipes like *chaise, Baidu roti* etc are prepared from the grains (40–50/persons); Paste prepared by grinding of (50 g/person) seeds with water applied on the fractured part
39	*Vigna unguiculata* (L.) Walp {Fabaceae (RKT 16856)}		Cowpea	*Lobia/Sotta/Sunt*	S, M, W	C	Diabetes and energy booster	Co	Taken as a traditional dish form (40–50 g/person)
40	*Macrotyloma uniflorum* (Lam.) Verde {Fabaceae (--)}		Horsegram	*Gahat*	S, M, W	C	Kidney stone, cold, and cough	Co	Pulse (*Daal–*40–50 g/person) soup; *Gahat ka Ras* (an indigenous dish) prepared by seeds (50 g/individual) cooked with water (1 ltr.) until the volume reduced (100 ml) and taken regularly
41	*Vigna umbellata* (Thunb.) Ohwi & Ohashi {Fabaceae (--)}		Rice bean	*Guruns/Rayans*	W	C	Jaundice and measles	Co	Pulse (*Daal*) of grains (50 g/individual)
**D**	Fruits								
42	*Citrus hystrix* DC. (Rutaceae) {Rutaceae (--)}		Jambhiri lemon	*Jamir*	W	C	Malaria, fever, and dehydration	Sy	Concentrated juice or extract (5–10 ml.) of matured fruits
43	*Citrus limon (L.)* Burm. f. {Rutaceae (RKT 2381)}		Lemon	*Nimbu*	W	C	Vitamin C, vomiting, and gastric disorder	In, Sy	Fruit taken as traditional nimbu *sanni*; fruit juice, or extract (5–10 ml) is ingredients of traditional chutneys
44	*Ficus palmata* Forssk. {Moraceae (RKT 8886, RKT 28094)}		Fig	*Bedu*	S, M	Wd	Gastric ulcer, cuts and wounds, and removal of thorn	Co, DA	Vegetables of fruits (50 g) for gastric ulcer; stem latex (4–5 drops) for removal of thorn form the bottom of the feet.
45	*Ficus auriculata* Lour. (Moraceae) {Moraceae (RKT 7524)}		Elephant ear fig	*Timila*	S, M	Wd	Acidity, blood pressure, and duodenal ulcer	Co	Vegetables of fruits (50 g/individual)
46	*Punica granatum L.* {Lythraceae (RKT 28845)}		Pomegranate	*Darim*	S, M, W	C, Wd	Anemia, cold and cough, and source of vitamin ‘C'	Po, In	Fruit peels (5–10 g) used for 3–4 times or powder (50 g) of dried fruit peel taken orally with warm water for cold and cough; fruit juice (50 ml) given twice a day to anemic patient
47	*Psidium guajava* L. {Myrtaceae (RKT 13868)}		Guava	*Amrood*	W	C	Cold and cough and mouth blisters	In	Fruits (50 g) baked in the hot ash are served to the patients; fresh leaves are chewed as astringent
48	*Syzygium cumini* (L.) Skeels {Myrtaceae (RKT 8839)}		Blackberry	*Jamun*	S	Wd	Diabetes	In	Fruits and seeds (100 g)
**E**	Spices and condiments								
49	*^a^* *Allium cepa* L. {Alliaceae (RKT 7675)}		Onion	*Pyaz*	S, M, W	C	Pimples	O	Paste of bulbs (50 g) applied on the spot
50	*^a^* *Allium sativum* L. (Alliaceae) {Alliaceae (RKT 19219)}		Garlic	*Lahsun*	S,M,W	C	Gastric problem and joint pain (Arthritis)	In, O	Cloves (2–3 nos.) are eaten in the morning before breakfast; paste prepared from 5 to 7 spilled cloves heated with 20 ml mustard oil and massage on joints
51	*Allium schoenoprasum* L. {Alliaceae (RKT 24974)}		Chives	*Dunn/Dhungar/Panguri*	S,M,W	C	Cold and cough, gastric problem, and joint pain	Co	Soup of cloves (20 g) and fresh (20 g) and dried leaves (20 g) taken as a vegetable; tempering the dishes
52	*^a^* *Capsicum annuum* L. {Solanaceae (RKT 7675)}		Chilli	*Khursani*	S, M, W	C	Skin burn	O	Paste of powder of chilli capsules applied over burned parts
53	*Cannabis sativa* L. {Cannabaceae (RKT 27224)}		Hemp	*Bhang*	S, M, W	C	Constipation, stomach ache, and warm effect	Co	Seed milk (100 ml) extracted from 20 g seed and used as an ingredient of traditional dishes
54	*^a^* *Coriandrum sativum* L. {Apiaceae (RKT 29354, RKT 28118)}		Coriander	*Dhania*	S, M, W	C	Urine disorder	Inf	Coriander grains (20 g) are soaked in water (100 ml); this water is served to the patients
55	*^a^* *Trachyspermum ammi* (L.) Spr. {Apiaceae (--)}		Ammi	*Ajwain*	S, M	C	Stomach ache and gastric problem	In, Po	Seeds (5 g) are chewed or powder is consumed with lukewarm water
56	*^a^* *Curcuma longa* L. (Zingiberaceae) {Apiaceae (RKT 5970)}		Turmeric	*Haldi*	S, M, W	C	Cuts, internal injury, and wounds	O, Po, DA	Paste of rhizomes for cuts and wound healing; powder (5 g) mixed with a full glass of warm milk for internal injury; dry leaves (5–10 nos.) are used as bed for infants
57	*Trigonella foenum–*graecum L. {Fabaceae (RKT 29342, RKT 28507)}		Trigonella	*Methi*	S, M, W	C	Cold and cough, constipation, diabetes, indigestion, joint pain, and obesity	Co, Inf	Vegetable of grains (12 g/individual) and leaves (50 g/individual); leaf juice (5–10 ml) is taken orally for curing obesity, indigestion, joint pain and constipation; 25 g seeds are soaked overnight and filtered, the filtrate taken orally on empty stomach for gastric problems and diabetes
58	*^a^* *Zingiber officinale* Roscoe {Zingiberaceae (RKT 5921)}		Ginger	*Adrak*	S, M, W	C	Cold and cough and viral fever	In, Sy	Baked rhizomes and soup (50 g) or a piece (5–10 g) of broiled rhizome mixed with small amount of honey and chewed
**F**	Oilseeds								
59	*^a^* *Brassica campestris* L. {Brassicaceae (RKT 26286)}		Yellow mustard	*Pili sarson*	S, M, W	C	Paralysis treatment	O	Oil (100 ml) massage on the affected parts
60	*^a^* *B. juncea* (L.) Czern {Brassicaceae (RKT 2286)}		Brown mustard	*Bhuri sarson*	S, M, W	C	Paralytic limbs	O	Oil (100 ml) massage on the affected parts
61	*Brassica nigra* (L.) Koch {Brassicaceae (--)}		Black mustard	*Raituwa rai*	S, M, W	C	Stomachic, refreshing agent, and relieves tiredness	In	Ground seed (5 g for 500 ml curd) is a main ingredients of traditional *Pahari Raytas*
62	*Lepidium sativum L*. (Brassicaceae) {Brassicaceae (--)}		Pepper cress	*Chamsur, Halang*	W	C	Asthma, cold and cough, and massage (infant)	Co, O	Leafy vegetables (30 g) for cold cough; fatty oil (8–10 drops) body massage for infant
63	*Linum usitatissimum* L. {Linaceae (RKT 22549)}		Linseed	*Alsi*	S, M, W	C	Immunity enhancer, and joint pain	DA, Po, In	Seeds or seed powder (1 g/day) taken early in the morning; Roasted seeds (25 g/individual) is an ingredient for traditional chutneys
64	*Perilla frutescens* (L.) Britton {Lamiaceae (RKT 28724)}		Perilla	*Bhangira*	S, M, W	C	Cough and joint pain	In	Roasted seeds (20g/individual) are an ingredient for traditional chutneys
65	*Ricinus communis* L. {Euphorbiaceae (RKT 22941)}		Castor	*Arandi*	S, M, W	Wd	Vein and artery problems	O	Oil (10–20 drops) massage
66	*Sesamum indicum* L. {Pedaliaceae (RKT 25193)}		Sesame	*Til*	S, W	C	Washing hair, cold and cough, and muscular pain	DA, In	Crushed green leaves (20–25 g) are used as shampoo; dried seeds (100 g) or crushed seed powder mixed with jaggery, small balls are prepared, and consumed during the winter season as medicine, Oil used in massage
**G**	Aromatic plants								
67	*Mentha arvensis* L. {Lamiaceae (RKT 1838; RKT 4355)}		Mint	*Pudina*	S, M	C	Diarrhea	In	Sauces (*Chutney*) of young tender leafy stem leaves and twigs (10–20 g)
68	*Ocimum basilicum* L. {Lamiaceae (RKT 1836, 19325)}		Basil	*Tulsi*	S, M	C	Cold and cough and viral fever	Inf	Tea of leaves or soup (10–15 nos. of leaves) of boiled leaves

a Eaten on a daily basis; ^$^Availability: crop season, storage, and market; S=summer (April to June); M = monsoon (July to September); Winter (December to March); cultivated (C); wild (Wd); ointment (O); ingestion (In); infusion (Inf); powder (Po); direct application (DA); cooking (Co); syrup (Sy); ash form (AF); smoke (S). #Matched vouchers with the herbarium specimen lodged in CCRAS-RARI, tarikhet, Ranikhet, Uttarakhand with the acronym (RKT).

The main characteristics of traditional farming are that it is performed on small and fragmented land holdings and is subsistence-type and labor-intensive, therefore practiced as family farming. It is largely organic in nature as the community uses only farmyard manure (FYM) to maintain soil fertility. The traditional food system comprised a combination of food plants, wild edibles, and other cuisines, etc., and there was a preference for traditional food items/dishes that maintained taste, nutrition, and cultural values. Women are at the center of the traditional food systems that undertake diverse roles from seed selection, storage, agriculture field preparation, weeding, harvesting, storage, cooking, etc., protecting farm diversity and maintaining dietary diversity within the community. The community strongly believed that the traditional food items substantiate their energy, protein, and carbohydrate requirements, particularly of women who performed extensive physical works at households and farms. An assessment of average food self-sufficiency revealed that communities would produce a significant share of their household need from their farms. For generations, the community has been successful in extending proper local governance in a decentralized manner to various resources, such as land, forest, and water, on which the traditional food system is intensely reliant. All these resources form an integral part of the local socioecological systems. All natural resources are maintained with the perspectives for proper access, harvest cycles, and equitable distribution, which is key to sustaining local food systems and diets.

### Seasonal Dietary Analysis

It was noticed that the dietary habits of the local community are influenced by seasons as per the availability of crops and wild plants. Some seasonal food items are also brought to the local market, and people can procure them from the market if it is not grown by them. An assessment of 24-h dietary recall of the community against the recommended dietary allowance (RDA) indicated that a large share of daily energy requirements of a working woman was fulfilled by traditional diets only (Figure 2). The community prefers energy-rich foods in their diets. An assessment of energy requirement of the age group of 19–50 years against the RDA revealed that traditional crops fulfill 78.97% requirement during summer, 73.29% during monsoon, and 92.88% in winter (Tables 2, 3). The energy intake was higher during winter than in monsoon. During winter, the community consumes more energy-rich foods such as *Eleusine coracana*, *Dioscorea bulbifera*, *Glycine max*, and *Amaranthus caudatus* (Table 4). Protein intake was recorded as 60.90 g/day in summer, 61.73 g/day in monsoon, and 77.85 g/day in winter, which is higher than the recommended intake; however, it can be attributed to the amount of labor performed for various works. Fat intake was confined within the RDA limits that reduce the risk of cholesterol-linked diseases. The traditional food also supports higher carbohydrate content in winter (345 g/day) than summer (298 g/day) and monsoon (277 g/day). Traditional food supports a higher intake of vitamin D within tolerable limits. Occasional consumption of meat and fish products also met nutrient demands. A low intake of calcium and sodium was observed, although the requirement of the latter element is met from natural salts. The study clearly revealed that traditional food crops are an important asset to food and nutritional security even in remotely located villages.

**TABLE 2:** Mean daily, weekly, and rare intake of food crops in different seasons.

Consumed common food crops	Parts used/consumed	^a^Average intake (g/day/individual)			
		Summer		Monsoon	Winter
Daily basis					
*Allium cepa* L	Bulb		69.00 ± 19.27	61.00 ± 10.99	57.00 ± 12.00
*Allium sativum*L	Bulb (cloves)		04.00 ± 2.20	11.00 ± 4.36	25.00 ± 08.46
*Brassica juncea* (L.) Czern	Seed oil		30.00 ± 06.89	32.00 ± 06.22	34.00 ± 08.03
*Capsicum annuum* L	Fruit capsule		08.00 ± 02.85	08.00 ± 02.56	12.00 ± 06.25
*Coriandrum sativum* L	Seed		24.00 ± 06.97	20.00 ± 07.54	20.00 ± 06.70
*Cucumis sativus* L	Fruit		105.0 ± 17.90	72.00 ± 20.36	--
*Curcuma longa* L	Rhizome		05.00 ± 03.41	05.00 ± 03.11	7.00 ± 02.95
*Oryza sativa* L	Seed (Grains)		190.00 ± 16.24	135.00 ± 17.03	160.00 ± 09.97
*Solanum tuberosum* L	Stem		75.00 ± 7.15	75.00 ± 11.12	90.00 ± 21.20
*Trachyspermum ammi* (L.) Spr	Seed		05.00 ± 0.82	05.00 ± 0.88	10.00 ± 0.83
*Triticum aestivum* L	Seed (grains)		150.00 ± 10.33	140.00 ± 18.54	100.00 ± 21.73
*Zingiber officinale* Roscoe	Rhizome		10.00 ± 03.82	15.00 ± 04.05	15.00 ± 04.90
Weekly (twice in a week)					
*Allium schoenoprasum* L	Cloves and leaf		10.00 ± 05.20	--	20.00 ± 05.98
*Amaranthus caudatus* L. (Amaranthaceae)	Seed (grains)		--	15.00 ± 12.62	60.00 ± 26.64
*Brassica oleracea* var. *capitata* L	Vegetative buds		--	--	65.00 ± 28.49
*Chenopodium album* L	Leaf twig		--	--	30.00 ± 12.84
*Cucurbita moschata* Duch. ex Poir	Fruit		--	100.00 ± 44.60	60.00 ± 32.13
*Cyclanthera pedata* (L.) Schrad	Fruit		--	130.00 ± 55.04	--
*Colocasia esculenta* L	Corms and petiole		--	80.00 ± 38.54	120.00 ± 60.97
*Dioscorea bulbifera* L	Aerial tuber		--	--	130.00 ± 28.65
*Eleusine coracana* (L.) Gaert	Seed (grains)		--	70.00 ± 18.86	110.00 ± 31.45
*Fagopyrum esculentum* Moench	Leaf		75.00 ± 28.67	--	--
*Glycine max* (L.) Merrill	Seed		38.00 ± 14.09	33.00 ± 11.11	50.00 ± 11.72
*Glycine max* subsp*. soja* (Sieb. & Zucc.) H. Ohashi	Seed		40.00 ± 23.30	35.00 ± 15.13	60.00 ± 31.22
*Lagenaria siceraria* (Molina) Standl	Fruit		80.00 ± 19.55	95.00 ± 21.82	--
*Lens culinaris* Medik	Seed		30.00 ± 18.47	35.00 ± 16.39	55.00 ± 30.53
*Luffa acutangula* (L.) Roxb	Fruit		85.00 ± 20.99	105.00 ± 23.42	--
*Macrotyloma uniflorum* (Lam.) Verde	Seed		25.00 ± 10.83	30.00 ± 11.26	75.00 ± 20.24
*Momordica charantia* L			--	50.00 ± 27.59	--
*Phaseolus vulgaris* L	Seed		45.00 ± 23.55	40.00 ± 20.01	70.00 ± 31.34
*Raphanus sativus* L	Whole plant		50.00 ± 9.5	65.00 ± 10	70.00 ± 8.4
*Spinacia oleracea* L	Leaf		--	--	100.00 ± 45.41
*Trichosanthes anguina* L	Fruit		--	70.00 ± 13.62	--
*Trigonella foenum–graecum* L	Seed and leaf		14.00 ± 02.47	12.00 ± 03.02	^b^50.00 ± 11.12
*Vigna mungo* (L.) Hepper	Seed		30.00 ± 16	35.00 ± 18	55.00 ± 30
*Vigna unguiculata* (L.) Walp	Seed		25.00 ± 16	45.00 ± 22	50.00 ± 30
Occasionally used (taken only on seasonal basis)					
*Asparagus filicinus* Buch.-Ham. ex D. Don	Tender shoots		70.00 ± 36.12	--	--
*Bauhinia variegata* L	Flower buds		10.00 ± 2.5	--	--
*Benincasa hispida* Thunb	Fruit		--	35.00 ± 17.41	--
*Brassica nigra* (L.) Koch	Seed		5.00 ± 2.53	5.00 ± 2.52	5.00 ± 4.20
*Cannabis sativa* L	Seed		10.00 ± 8.34	10.00 ± 7.27	20.00 ± 11.95
*Citrus hystrix* DC.	Fruit		--	--	90.00 ± 39.40
*Citrus limon* (L.) Burm. f	Fruit and extract		10.00 ± 7.14	10.00 ± 7.49	90.00 ± 44.03
*Dioscorea alata *L	Tuber		--	--	50.00 ± 29.39
*Diplazium esculentum* (Retz.) Sw	Young fronds		40.00 ± 22.95	40.00 ± 22.20	--
*Echinochloa frumentacea* Link	Seed (grains)		50.00 ± 29.39	--	50.00 ± 29.61
*Fagopyrum esculentum* Moench	Seed (grains)		40.00 ± 23.87	--	40.00 ± 24.13
*Fagopyrum cymosum* Trev	Leaf		70.00 ± 38.56	--	--
*Ficus palmata* Forssk	Fruit		35.00 ± 18.46	40.00 ± 20.44	--
*Ficus auriculata* Lour	Fruit		45.00 ± 27.46	53.00 ± 29.40	--
*Hordeum vulgare* L	Seed (grains)		20.00 ± 11.07	17.00 ± 08.95	--
*Lepidium sativum* L	Leaf		--	--	30.00 ± 12.84
*Linum usitatissimum* L	Seed		20.00 ± 12.58	20.00 ± 11.95	35.00 ± 21.37
*Megacarpaea polyandra* Benth	Leaf		70.00 ± 36.91	--	--
*Mentha arvensis* L	Leaf		20.00 ± 08.31	15.00 ± 07.80	--
*Ocimum basilicum* L	Leaf		08.00 ± 04.50	06.00 ± 03.29	--
*Perilla frutescens* (L.) Britton	Seed		20.00 ± 13.07	10.00 ± 5.03	20.00 ± 11.60
*Phytolacca acinosa* Roxb	Leaf		50.00 ± 29.39	40.00 ± 22.95	--
*Psidium guajava* L	Fruit		--	--	50.00 ± 22.46
*Punica granatum* L	Fruit		20.00 ± 12.14	50.00 ± 22.42	30.00 ± 13.31
*Sesamum indicum* L	Seed		10.00 ± 06.08	--	15.00 ± 08.79
*Setaria italica* (L.) P. Beauv	Seed		80.00 ± 37.88	68.00 ± 29.94	50.00 ± 23.80
*Solanum melongena* L	Fruit		80.00 ± 36.63	85.00 ± 42.56	--
*Syzygium cumini* (L.) Skeels	Fruit		30.00 ± 13.72	--	--
*Urtica ardens* Link	Leaf twig		--	--	50.00 ± 29.39
*Vigna umbellata* (Thunb.) Ohwi& Ohashi	Seed		--	--	50.00 ± 29.66
*Zea mays* L	Seed		--	65.00 ± 32.14	--

a Average intake as per availability of the consumed part in different seasons.

b Used and consumed part only leaf; (**--**) no intake during respective season.

**TABLE 3:** Nutritional requirements and uptake from traditional food crops commonly eaten by the communities.

Nutrients	^a^RDA (per day)	^$^TUL	Daily and weekly average intake of nutrients in different seasons											
			Summer				Monsoon				Winter			
			Daily	Weekly	Total	(%)^b^FRDA	Daily	Weekly	Total	(%)*FRDA	Daily	Weekly	Total	(%)*F RDA
Energy (KCal)	2,200.00	--	1,452.92	284.41	1737.33	78.97	1,225.08	387.31	1,612.39	73.29	1,223.56	819.69	2043.25	92.88
Protein (g)	46.00	--	44.16	16.74	60.90	132.39	38.77	22.95	61.73	134.19	38.31	39.55	77.85	169.24
Fat (g)	28.00	--	17.78	3.48	21.26	75.91	17.97	5.47	23.44	83.70	19.74	10.22	29.95	106.98
Carbohydrate. (g)	130.00	--	274.64	23.87	298.51	229.62	223.19	53.51	276.69	212.84	224.40	120.66	345.06	265.43
Vit. B1 (mg)	01.10	ND	1.07	0.25	1.32	119.66	1.00	0.38	1.38	125.62	0.87	0.65	1.53	139.06
Vit. C (mg)	75.00	2000.00	101.23	27.86	129.09	172.12	64.98	41.26	106.24	141.66	77.91	79.78	157.69	210.25
Vit. D (µg)	15.00	100.00	18.86	5.22	24.07	160.50	18.80	19.06	37.86	252.41	18.65	21.81	40.45	269.70
Vit. E (mg)	15.00	1,000.00	3.39	0.33	3.71	24.75	3.60	0.59	4.19	27.93	3.69	1.33	5.02	33.50
Ca (mg)	1,000.00	2,500.00	386.59	145.59	532.17	53.22	295.31	289.71	585.03	58.50	304.65	757.67	1,062.33	106.23
Fe (mg)	18.00	45.00	17.87	14.44	32.31	179.52	16.52	8.37	24.89	138.28	14.94	16.94	31.88	177.12
Mg (mg)	320.00	350.00	222.33	103.21	325.55	101.73	187.61	167.14	354.76	110.86	193.11	297.59	490.71	153.35
Na (mg)	1,500.00	2,300.00	55.86	17.84	73.71	4.91	53.70	20.95	74.65	4.98	42.05	84.56	126.62	8.44
Zn (mg)	08.00	40.00	8.44	2.31	10.75	134.38	7.34	3.47	10.82	135.22	6.81	6.92	13.73	171.58

a Recommended dietary allowance (RDA); tolerable upper limit (TUL).

b Fulfillment against RDA; ND- not determined.

**TABLE 4:** Key nutrient-rich food crops used in traditional diets.

Use purpose	Key sources in traditional diet (crop species)
Energy	*Lepidium sativum* L.*, Macrotyloma uniflorum* (Lam.) Verde, *Setaria italica* (L.) P. Beauv., *Eleusine coracana* (L.) Gaert., *Glycine max* (L.) Merrill, *Vigna umbellata* (Thunb.) Ohwi & Ohashi, *Brassica juncea* (L.) Czern, *Triticum aestivum* L., and *Fagopyrum esculentum* Moench
Protein	*Amaranthus caudatus* L.*, Brassica juncea* (L.) Czern, *Glycine max* (L.) Merrill, *Glycine max* subsp. *soja* (Sieb. & Zucc.) H. Ohashi, *Lepidium sativum* L.*, Sesamum indicum* L., and *Trigonella foenum–graecum* L
Fat	*Sesamum indicum* L., *Glycine max* (L.) Merrill, *Trachyspermum ammi* (L.) Spr., *Brassica juncea* (L.) Czern, and *Glycine max* subsp. *soja* (Sieb. & Zucc.) H. Ohashi
Carbohydrate	*Amaranthus caudatus* L.*, Dioscorea bulbifera* L., *Oryza sativa* L., *Setaria italica* (L.) P. Beauv., *Zea mays* L., *Hordeum vulgare* L., *Solanum tuberosum* L., *Phaseolus vulgaris* L., *Vigna unguiculata* (L.) Walp, and *Colocasia esculenta* L
Vitamin B1	*Lepidium sativum* L., *Sesamum indicum* L., *Glycine max* (L.) Merrill, *Vigna mungo* L., *Vigna unguiculata* (L.) Walp, and *Phaseolus vulgaris* L
Vitamin C	*Allium cepa* L., *Capsicum annuum* L., *Raphanus sativus* L.) Hook, *Citrus hystrix* DC, *Citrus limon* (L.) Burm. f., *Fagopyrum esculentum* Moench, and *Brassica oleracea* var. *capitata* L
Vitamin D	*Amaranthus caudatus* L.*, Glycine max* (L.) Merrill, *Punica granatum* L., *Brassica juncea* (L.) Czern., *Eleusine coracana* (L.) Gaert, *Vigna mungo* (L.) Hepper, *Trichosanthes anguina* L., and *Cucurbita moschata* Duch. ex Poir
Vitamin E	*Brassica juncea* (L.) Czern, *Curcuma longa* L. and *Vigna umbellata* (Thunb.) Ohwi & Ohashi

### Dietary Diversity Scores

An investigation of the household dietary diversity score (HDDS) was also undertaken to assess the households’ access to a variety of foods (Table 5). It was interesting to note that most households were consuming adequate nutritional food in all the seasons, and none of them were placed with low dietary scores. The HDDS ranged from 4 to 10, being maximum in winter, followed by summer and monsoon months. The communities were fed well in all the food groups with daily consumption of cereals, oils and fats, vegetables, and milk products as well as meat and related products in all the seasons. The consumption of meat products was low in monsoon. There was low consumption of sugar edibles and fruits in almost all the seasons, although vitamin C–rich fruits were consumed in ample quantity in winter.

**TABLE 5:** Dietary diversity at the household level in central Himalaya.

Food groups (12-FGI)	Household dietary diversity distribution					
	Summer (HDDS = 7.45)		Monsoon (HDDS = 7.34)		Winter (HDDS = 8.39)	
	MDS (4–5) (*n* = 11)	HDS (>6) (*n* = 89)	MDS (4–5) (*n* = 10	HDS (>6) (*n* = 90)	MDS (4–5) (*n* = 8)	HDS (>6) (*n* = 92)
Cereals (%)	100.00	100.00	100.00	100.00	100.00	92.00
White roots & tubers (%)	54.55	98.88	60.00	100.00	62.50	90.00
Vegetables (%)	100.00	96.63	40.00	97.78	75.00	87.00
Fruits (%)	-	22.47	20.00	32.22	0.00	61.00
Meat (%)	-	53.93	-	24.44	12.50	40.00
Eggs (%)	-	34.83	-	31.11	-	48.00
Fish and other sea food (%)	-	16.85	-	-	-	39.00
Legumes, nuts, and seeds (%)	-	47.19	10.00	67.78	25.00	57.00
Milk and milk products (%)	36.36	98.88	60.00	94.44	25.00	83.00
Oil and fats (%)	72.73	100.00	90.00	100.00	100.00	91.00
Sweets (%)	-	6.74	-	14.44	12.5.00	19.00
Spices and condiments (%)	90.91	98.88	100.00	100.00	100.00	91.00

Medium dietary score (MDS); High dietary score (HDS).

The average nutrition adequacy ratio (NAR) for all the nutritional parameters was 9.51 with a mean adequacy ratio of 0.73, which promises good quality of food intake by the communities living in remote locations. Although the anthropometric was not undertaken, it was recorded that the community was not eating junk food, sugar additives, and other unhealthy dietary markers. It reduces the chances of obesity, diabetes, and other similar ailments. The population is found taking better intake of nutrition that can be an indicator of low risks of diabetes and other ailments as well.

**FIGURE 3:**
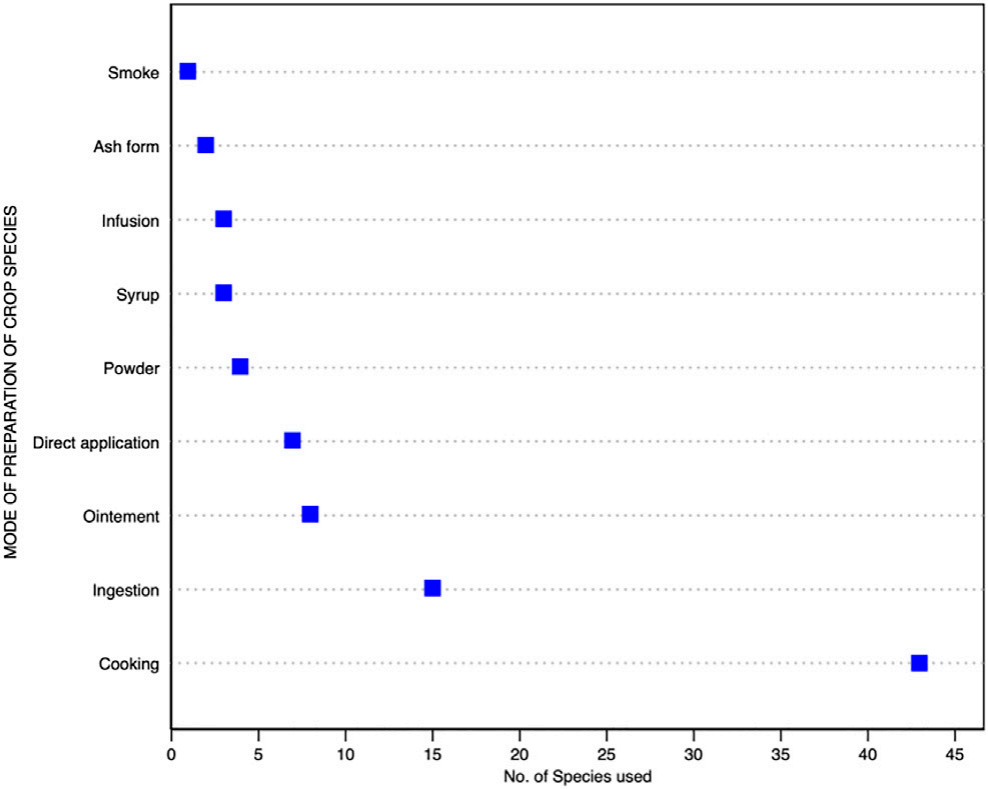
Species-specific methods in terms of their preparation for health care.

**FIGURE 4:**
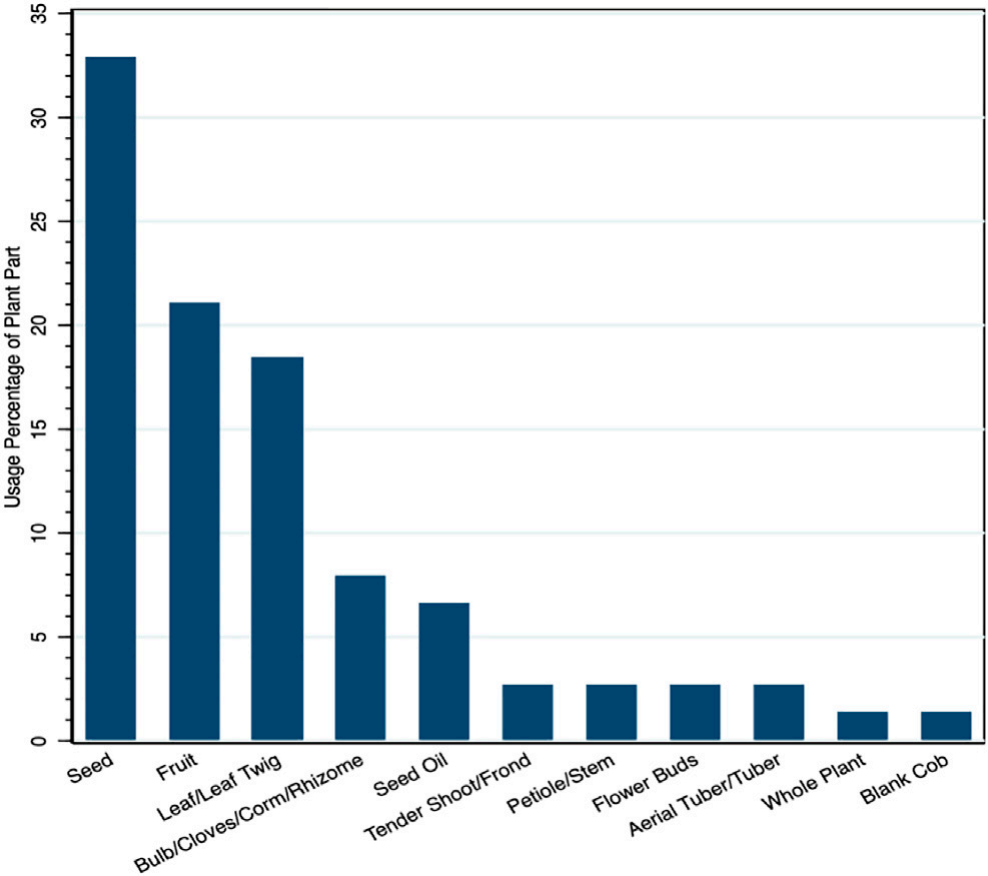
Plant part used in preparation of medicine.

**FIGURE 5:**
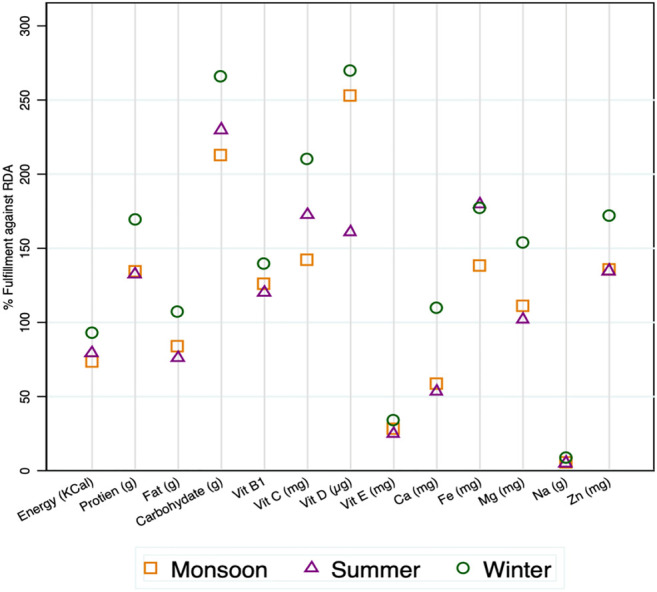
Seasonal variation in fulfillment of nutrition against the RDA.

It was interesting to note that traditional diets form a rich source of nutrients (Table 4). The most common mode to use species is in the form of cooked, direct ingestion, as an ointment, and applying it directly either by crushing, powder, or adding the part as it is (Figure 3). In terms of plant parts used for treating the ailments, they include seeds (32.89%), fruits (21.05%), and leaves (18.41%) followed by bulbs, seed oils, and others, etc. (Figure 4). Most species had multipurpose uses ensuring food and nutritional security as well as medicinal efficacy for curing minor ailments (Figure 5). The community had species-specific knowledge on the use and application of various species. A total of 43 species were reportedly used as dietary supplements and food additives with definite physiological benefits during different ailments. Such species are commonly consumed as cooked food; thus, it can be categorized as neutraceutical or bioceutical. Other common methods comprised ingestion (15 species), ointment (eight species), and direct application (seven species).

**FIGURE 6:**
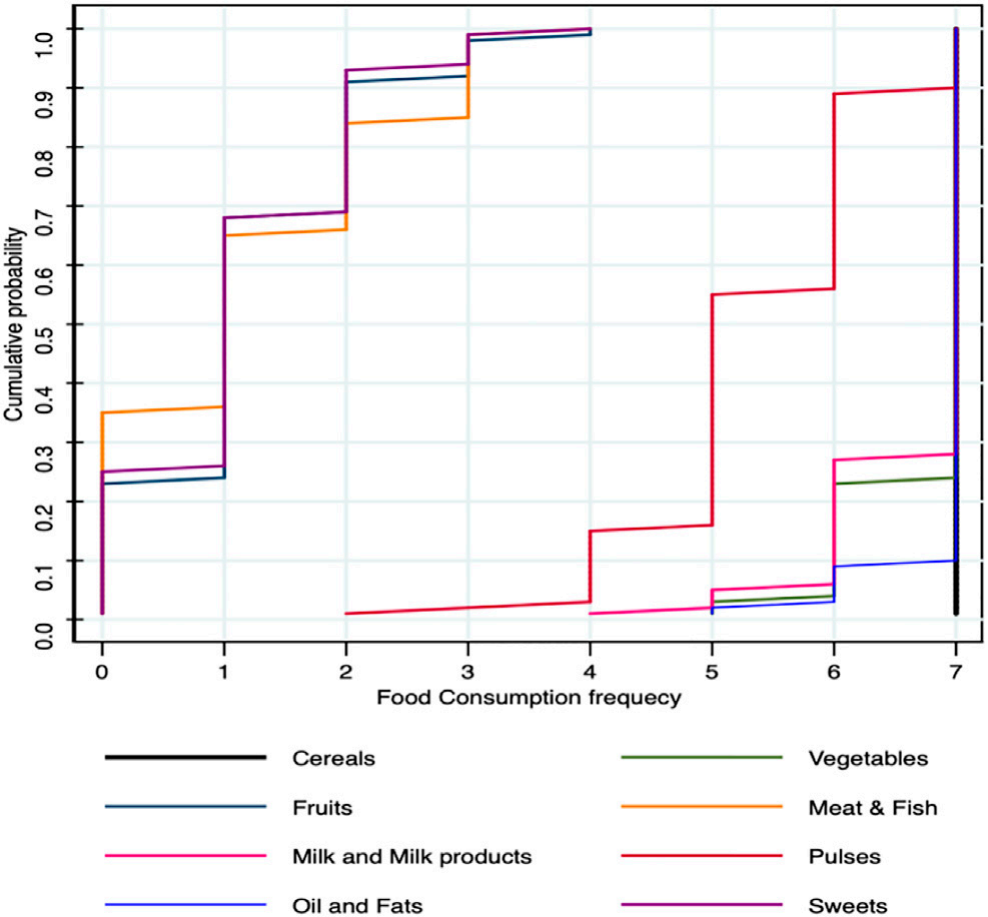
Cumulative probabilty of the FCS.

The average food consumption score of the communities was 73.46, which is a good sign of food diversity intake among communities ranging from a minimum score of 55.55 and a maximum score of 87.00. The average cumulative probability for the entire crop species intake in the FCS was above 0.6, showing higher diversity of intake (Figure 6).

### The Interface of Food and Medicine

Traditional food plants are rich sources of nutrients and chemical compounds that are used by the body to function properly and maintain health. An assessment of the nutritional status of crops plants revealed the presence of diverse components, such as proteins, fats, carbohydrates, vitamins, and minerals, in traditional foods and diets (Supplementary Annexure 1). Many species comprised high energy content (>3.5–5.5 kcal/g), such as S*esamum indicum*, *Trachyspermum ammi*, *Oryza sativa*, *Cannabis sativa*, *Brassica juncea*, *Glycine max* subsp. *soja*, *Lepidium sativum*, *Linum usitatissimum*, *Echinochloa frumentacea*, *Bauhinia variegata*, and *Glycine max*. While *Zea mays, Vigna mungo*, *Triticum aestivum*, *Setaria italica*, *Phaseolus vulgaris*, *Fagopyrum esculentum*, *Perilla frutescens*, *Vigna unguiculata*, *Vigna umbellata*, *Trigonella foenum–graecum*, and *Macrotyloma uniflorum* exhibits medium energy content (2.5–3.5 kcal/g), *Asparagus*, *Bauhinia variegata*, *Benincasa hispida*, *Brassica campestris*, *Brassica juncea*, *Brassica nigra*, *Brassica oleracea*, *Cannabis sativa*, *Capsicum annuum*, and *Chenopodium album* exhibited high protein content (>20–43 g/100 g). Similarly, *Cannabis sativa*, *Perilla frutescens*, *Sesamum indicum*, *Brassica juncea*, *Linum usitatissimum*, *Lepidium sativum*, and *Trachyspermum ammi* comprised high fat content (20–49 g/100 g). Many local species were also rich in vitamins and other nutrients (Supplementary Annexure 1). Thus, the data clearly reveal that the nutritional energy value (can be calculated as the sum of food energies of all components) of the traditional food systems was very high.

The use of plants and food recipes has been a fundamental component of all rural house treatment systems as it is the most easily accessible resource available to the local community at the time of medical urgency. The local communities are well aware of the potency of food crops and their utility for treating various ailments and illnesses. Although they may not be known for the scientific reason as to how these food crops work in the body in treating the ailment. Since such treatments have been in practice for centuries, they apply it as the best affordable means. A literature survey on traditional crops clearly exhibited high medicinal value ranging from antifungal, anti-inflammatory, decreasing risks of cancer, reducing the risk of diabetics, etc. (Supplementary Annexure 2). *Oryza sativa*, *Eleusine coracana*, *Glycine max*, *Vigna umbellata*, and *Macrotyloma uniflorum* were good sources of carbohydrates, while *Brassica juncea*, *Sesamum indicum*, and *Lepidium sativum* provided worthy fat sources. Some species also exhibited aphrodisiac, antimicrobial (fungal and bacterial), antidiabetic, cardioprotective, anticancer, analgesic, antianemic, hepatoprotective, immunomodulatory, swelling & cholesterol-reducing, blood sugar lowering, etc. characteristics. It clearly revealed that local food has enough provisions for appropriate diets to satisfy the nutritional and energy needs along with a good balance of vitamins and micronutrients to the local community. It is essential to perform diverse functions, such as from boosting the immunity to therapeutic actions and repair of cellular damage to the healing of wounds and ailments, thus acting as protective shields against different ailments.

An important aspect of the interface of food and medicine is the use of diverse food recipes and the community’s hyperawareness of its potency for curing different illnesses (Table 6). The use of food recipes varies with seasons. The mode of preparation, ingredients, cooking process, and intake frequency differs. The community uses foods with warm potency in monsoon and winter. Such species are *Amaranthus virdis*, *Chenopodium album*, *Dioscorea bulbifera*, *Hordeum vulgare*, *Eleusine coracana*, and *Macrotyloma uniflorum*, while, cool potency food such as *Cucumis sativus*, *Coriandrum sativum*, *Oryza sativa*, and *Raphanus sativus* were taken usually in summer. Several other criteria also work in selecting the food, such as people prefer eating meat products and coarse grain cereals more at higher altitudes due to cold. Other than achieving wellness, the traditional recipes also help address seasonal health issues. There is significant scope to prepare many new food dishes from traditional recipes to attract the market.

**TABLE 6:** Traditional recipes with major ingredients and its special uses in the central Himalaya.

	Name of traditional recipes (*vernacular name*)	Common name and ingredients	Important uses	
			As food	As medicinal purposes
**A**	**Chapati (bread)**			
1	*Chaulai ki roti*	Amaranth seed flour	SF, TF	IE, SE
2	*Jhangora/Madira ki roti*	Barnyard millet flour	SF	SE
3	*Kutu ki roti*	Buckwheat flour	SF, TF	SE
4	*Kauni ki roti*	Foxtail millet flour	SF	SE
5	*Madua ki roti*	Finger millet flour	SF, TF	IE, SE
6	*Makke ki Roti*	Maize flour	SF	SE
7	*Choi/Chhola roti*	Rice	SF	--
8	*Lesuwa roti*	Finger millet and Wheat flour	SF, TF	IE, SE
9	*Gahat ki bedu roti*	Horse Gram and wheat flour	SF, TF	IE, SE
10	*Gurunsh ki bedu roti*	Rice bean and wheat flour	SF	IE, SE
11	*Mash ki bedu roti*	Black Gram and wheat flour	SF	IE, SE
12	*Sonta (Lobia) ki bedu roti*	Cowpea and wheat flour	SF	IE, SE
**B**	**Rice**			
13	*Chaulai ka bhat*	Amaranth seeds	SF, TF	IE, SE
14	*Jhangora/Madira ka bhat*	Barnyard millet seed	SF	IE, SE
15	*Kauni ka bhat*	Foxtail millet seed	SF	Iln, IE, SE
**C**	**Dishes related to pulses**			
16	*Bhatia/Jaula*	Black or white soybean (bhatt) and rice	SF	AF, IE, SE
17	*Bhatt ke dubake*	Black soybean (kala bhatt)	SF	AF, IE, SE
18	*Bhatt ka fana*	Black soybean (kala bhatt) and finger millet flour	SF	AF, IE, SE
19	*Bhatt mathi ki sabzi*	Black/white soybean (bhatt) and methi leaves	SF	AF, IE, SE
20	*Bhatt papad ke sabzi*	Black/white soybean (bhatt) and dried petiole/stalk of Taro	SF	IE, SE
21	*Chudkani*	Black soybean (kala bhatt) and rice flour	SF, TF	AF, Iln, IE, SE
22	*Gahat ki dal*	Horse gram and heeng	SF, TF	Iln, SE
23	*Gahat ke dubake*	Horse gram	SF	Iln, SE
24	*Gahat ka fana*	Horse gram and finger millet flour	SF, TF	Iln, SE
25	*Gahat gaderi ki dal*	Horse gram and Taro	SF	Iln, SE
26	*Gahat muli ki sabzi*	Horse gram and radish	SF, TF	AF, Iln, IE, SE
27	*Maas/Urad ka chaisa*	Black gram	SF, TF	SE
28	*Maas/Urad ki dool dal* (Khari dal)	Black gram	SF, TF	SE
29	*Maas/Urad ke bade*	Black gram paste	SF	SE
30	*Masur dal ke sabzi*	Lentil paste and onion and Garlic	SF	Iln, SE
31	*Ras* (extract of mixed pulses)	(Horse gram, black or white bhatt, cowpea, black gram, gram, rice bean, French bean) and rice flour	SF, TF	AF, IE, SE
32	*Sonta (Lobia) ka chaisa*	Cowpea	SF	SE
33	*Sonta (Lobia) ke bade*	Cowpea paste	SF	SE
**D**	**Vegetables**			
34	*Aalo or muli ka thinchwani*	Potato or raphanus and hemp seed milk	SF, TF	SE
35	*Aalu muli ki sabzi*	Potato and radish and curd/buttermilk	SF, TF	SE
36	*Aalu sarson ki sabzi*	Potato and Mustard seeds	SF, TF	SE
37	*Bathua ki sabzi*	Tender twigs of *Chenopodium*	SF, TF	Iln, IE, SE
38	*Barmola/Rooki ki sabzi*	Rooki leaf and root	SF	AF, AS, Iln, IE, SE
39	*Bichhu/Kandali ka saag*	Soft twigs *Urtica* sp. and garlic and heeng	SF, TF	AF, AS, Iln, IE, SE
40	*Bhang aur gaderi ki sabzi*	Hemp seed milk and taro tuber	SF, TF	AS, IE, SE
41	*Bhang aur genthi ki sabzi*	Hemp seed milk and genthi yams (aerial tuber)	SF, TF	AF, AS, Iln, IE, SE
42	*Bhang aur gobi ki sabzi*	Hemp seed milk and cabbage	SF, TF	AS, SE
43	*Dhungar/Dunna ke sabzi*	*Allium sp*. and curd/buttermilk	SF, TF	AF, AS, Iln, IE, SE
44	*Genthi ki sabzi*	Dioscorea/yam (aerial tuber)	SF, TF	AF, AS, Iln, IE, SE
45	*Jarag ka saag*	Jarag tender twigs	SF, TF	Iln, SE
46	*Jhankara ka saag/Tinari*	Wild buckwheat tender twigs and leaves	SF	AF, AS, IE, SE
47	*Kairua ka saag*	Tender shoots of *Asparagus* sp	SF, TF	IE, SE
48	*Kafa/Kapa (Palak ka Kapa)*	Spinach and rice flour	SF, TF	IE, SE
49	*Karela ka bharuwa/sabzi*	Bitter gourd and fennel	SF	Iln, IE, SE
50	*Kewral/Gwaral ki sabzi*	Tender flower bud of *Bauhimia* sp	SF	Iln, IE, SE
51	*Lahsun ki sabzi*	Garlic	SF, TF	Iln, IE, SE
52	*Lingura ki sabzi Lingura*	*Lingura* fern fronds	SF	IE, SE
53	*Meetha/Ram karela ki sabzi*	Fruits of wild cucumber	SF	IE, SE
54	*Patyude/Pinalu ka gunuwa*	Leaves of taro and lentil or gram flour	SF	--
55	*Pindalu/Dharud ke gabe/sabzi*	Taro rolled leaf blade and petiole or leaf stalk and radish	SF	--
56	*Tarur ki sabzi*	Tuber of winged yam	SF	SE
57	*Timila ki sabzi*	*Ficus auriculata* tender fruits	SF	AF, AS, Iln, IE, SE
58	*Ogal/Phafar ka saag*	Buckwheat tender twigs and leaves	SF, TF	IE, SE
**E**	**Bari (prepared by mixing black gram bean flour with vegetables and)**			
59	*Bhuj ki bari ke sabzi*	Bari (wax gourd and black gram) and rice/wheat flour/gram flour	SF, TF	--
60	*Kakadi ki bari ke sabzi*	Bari (matured cucumber and black gram) and rice/wheat flour/gram flour	SF	--
61	*Mooli ki bari ke sabzi*	Bari (radish and black gram) and rice/wheat flour/gram flour	SF, TF	--
62	*Pinalu ki bari ke sabzi*	Bari (taro tuber and black gram) and rice/wheat flour/gram flour	SF	--
63	*Pinalu ke danthal/dhare ki bari*	Bari (taro petiole or leaf stalk and black gram) and rice/wheat flour/gram flour	SF	--
**F**	**Raita (prepared with curd)**			
64	*raita*	Cucumber and curd and brassica seed (rai)	VS	AF, AS
65	*Kewral/Gwaral ka raita*	Tender flower buds of *Bauhinia* and curd and brassica seed (rai)	VS	AF, AS, Iln
66	*Lauki ka raita*	Bottle gourd fruits and curd and brassica seed (rai)	VS	AF, AS, Iln
67	*Mooli ka raita*	Radish and curd and brassica seed (rai)	VS	AF, AS
68	*Timila ka raita*	*Ficus auriculata* tender fruits and curd and brassica seed (rai)	VS	AF, AS, Iln
**G**	**Chutney (sauce, ketchup, and seasoning)**			
69	*Alsi ki chutney*	Linseed and lemon extract	SC	AF, AS, IE
70	*Bhang ki chutney*	Hemp seed and lemon extract	SC, TF	AF, AS
71	*Bhangeera ki chutney*	*Perilla* seeds and lemon extract	SC	AF, AS, IE
72	*Bhatt ki chutney*	Black/brown seeded soybean (bhat) and lemon extract	SC	AF, AS, IE, SE
73	*Darim ki chutney*	Wild pomegranate seeds and lemon extract	SC	AF, AS
74	*Kaddu ke meethi Chutney*	Pumpkin and hemp seed and jaggery and lemon extract	SC	AF, AS
75	*Nimbu ke saani*	Lemon and hemp seeds and curd and jaggery	SC, TF	AF, AS
76	*Til ki chutney*	Sesame seeds and lemon extract	SC, TF	AF, AS, IE
77	*Timila ki Chatni/Sanni*	*Ficus auriculata* fruit and lemon extract and mustard seed	SC	AF, AS, IE
**H**	**Pakories** (fritters))			
78	*Ogal/Phaphar ki Pakori*	Buckwheat flour and potato	S, TF	SE
79	*Palak ki pakori*	Spinach leaf and gram flour	S	SE
80	*Jarag ki Pakori*	Jarag tender twigs and gram flour	S	SE
**I**	**Sweet dishes**			
81	*Chaulai ki kheer*	Amaranth seeds and milk	D, TF	IE, SE
82	*Chaulai ka halwa*	Amaranth seeds and milk/water	D, TF	IE, SE
83	*Jhangora/Madira ki kheer*	Barnyard millet seed and milk	D	SE
84	*Jhangora/Madira ka halwa*	Barnyard millet flour	D	SE
85	*Kauni ki kheer*	Foxtail millet seed and milk	D	SE
86	*Khir-Khaja*	Rice and milk	D	SE
87	*Lapsi/Leta/Rautti*	Wheat flour and curd	D, TF	SE
88	*Meetha bhat*	Rice and jaggery	D	--
89	*Ogal/Phaphar ka halwa*	Buckwheat flour	D, TF	SE
90	*Rot*	Wheat flour and butter and milk	D	--
91	*Singal, Puwe*	Rice or suji flour and milk and curd and butter	D	--
92	*Shaya*	Rice or suji flour and curd and milk and butter	D	Iln
93	*Swanl-Ladao*	Wheat flour; rice flour and water and butter and sesame	D	--
**J**	**Other dishes**			
94	*Chhachhiya/Jaula*	Rice and curd/buttermilk	SF	Iln, SE
95	*Laina Jaul*	Buttermilk/curd and rice	SF	Iln, IE, SE
96	*Sattu*	Barley; wheat; and finger millet	SF	AF, AS, Iln, IE, SE
97	*Vigaud*	Colostrum of buffalo or cow	S	AF, AS, Iln, IE, SE

Antifatigue (AF); antistress (AS); dessert (D); illness (Iln); immunity enhancing (IE); snacks (S); spicy cuisine (SC); stamina enhancing (SE); staple food (SF); thermogenic food (TF); vegetable substitute (VS).

The health status of Bageswar reveals that the children below 5 years are impacted with anemia, stranded growth, and are underweight. Similarly, the women of reproductive age are anemic with less bodyweight, thus influencing their lactation ability. A major reason for this is the lack of proteinaceous food and timely supply of tonics and other medicines. Fortunately, there are a large number of local food plants that can supplement such dietary needs of the community. For example, species such as *Eleusine coracana*, *Setaria italic*, *Glycine max*, *Amaranthus caudatus*, *Fagopyrum esculentum*, *Chenopodium album*, *Punica granatum*, *Vigna umbellata*, *Syzygium cumini*, *Spinacia oleracea*, and *Urtica ardens* are useful to avoid anemia. In addition, in case of stunted growth, consumption of *Eleusine coracana*, *Glycine max*, *Glycine soja*, *Setaria italica*, *Vigna umbellata*, *Brassica juncea*, *Fagopyrum esculentum*, and *Linum usitatissimum* is considered beneficial. Likewise, to enhance breast milk, consumption of *Eleusine coracana*, *Glycine max*, *Glycine soja*, *Phaseolus vulgaris*, *Spinacia oleracea*, *Amaranthus caudatus*, *Fagopyrum esculentum*, and *Brassica oleracea* var. *capitata* is advisable. There are also many protein-rich traditional recipes such as *Bhatia/Jaula*, *Bhatt ke dubake*, *Bhatt ka fana*, *Bhatt mathi ki sabzi*, *Bhatt papad ke sabzi*, *Chudkani*, and *Sonta (Lobia) ka chaise*. (Table 6). The traditional recipes can also help overcome anemic conditions by consuming *Chaulai ki roti*, *Jhangora/Madira ki roti*, *Kutu ki roti*, *Kauni ki roti*, *Madua ki roti*, *Sonta (Lobia) ki bedu roti*, *Bathua ki sabzi*, *Bichhu/Kandali ka saag*, *Barmola/Rooki ki sabzi*, *Jhankara ka saag/Tinari*, etc. There is a need to make people aware of these plants and dishes to consume them for treating such illnesses.

## Discussion

Access to healthy food is a major global challenge; a major reason for this is changing food systems and steady decline in traditional diets (FAO, IFAD, UNICEF, WFP and WHO, 2020). As traditional food systems are well-adapted to the local ecological, sociocultural, and economic setting, they are best placed to carry forward the nutritional and health security among the masses (Niketan et al., 2018). In many places, such traditional systems are still prevalent, and there is a need to highlight and endorse the health, nutritional, and therapeutic benefits of the traditional food system to revive it from disappearance. This study explored a Central Himalayan community of the Bageshwar district, Uttarakhand (India) that is highly marginal; however, it still exhibits a significant dependence on the traditional food system. The traditional farming practice is age-old and time-tested, which greatly helped maintain crop diversity along with ensuring food security. This study provided some valuable data in this regard:

i) The community uses as many as 68 food plants as part of their regular diet to fulfill their basic food needs, and the efficacy of the traditional food system can be judged from the perspectives of production, consumption, nutrition, and healing characteristics. The production perspective comprised a diversity of cereals, millets, vegetables, fruits, spices and condiments, medicinal plants, and meat sourced for food from farming or wild areas; the consumption perspective comprised the diversity of foods in local diets along with its cultural identity; the nutritional significance refers to the contents of food that fulfill the nutritional requirements, such as proteins, carbohydrates, fats, minerals, and vitamins, of the local community, thus minimizing nutrition deficiency in the community; while the healing perspective comprised remedial treatments offered by food and wild plants to accomplish medicinal and health security. This clearly reflects that the native dietary pattern fulfills diverse requirements of the marginal hill community, and a large section still considers it as a healthy food system.
ii) The communities possess significant knowledge about the local plants and diets (cultivation or wild collection, storage, food preparation, seasonal uptake, nutritional traits, therapeutic efficacy, etc.), which makes it a highly resilient food system. The foods support diverse and nutritionally rich diets in different seasons holistically and comprehensively. Continuity of such a system is greatly required to maintain on-farm crop diversity and species richness.
iii) The community has been successful for generations in maintaining the food production system embedded in the local sociocultural and ecological context that safeguards local communities to afford healthy diets at their home, thus providing an answer to counter malnutrition in the community.
iv) Women are at the core of the traditional food system that plays a key role in maintaining dietary diversity within the community. Their voices can lead to expansion of traditional agriculture and diversification of local diets for ensuring nutrient adequacy within indigenous territories.
v) The community has been successful in providing proper governance to various resources, such as land, forest, and water, on which the traditional food system has strong dependence. The community manages these resources as an integral part of the local livelihood in a socially acceptable and decentralized manner by maintaining access, harvest cycles, and equitable distribution, which is key to sustaining local food systems and diets.

Given the abovementioned situation, it is clear that traditional food systems, local knowledge, sociocultural setup, women, and resource governance together can lead to maintaining a traditional agroecological system that can provide a basis for a future of holistic food and health system. A large share of global crop genetic diversity including landraces and wild plants and animals is under the communities’ custody (Oldfield and Alcorn 1987). Traditional food systems are largely practiced by smallholders, particularly by women, who maintain local food habits and genetic diversity and soil fertility of agricultural fields (FAO 2011a; FAO 2011b; Sundriyal et al., 2014; Maikhuri et al., 2015). They are low-cost, energy-efficient, local resource–dependent, and climate-smart systems, thus contributing immensely to food supply and environmental sustainability (Sundriyal et al., 1994; CINE 2021). The dietary diversity index works as a proxy for nutritional security and varies in seasons, which is also a resemblance to our study (Hjertholm et al., 2019). Worldwide previous studies concluded that dietary diversity has a positive alliance with nutritional adequacy (Ruel, 2003; Steyn et al., 2006; Faber et al., 2009). The inclusion of a household healthy diet can reduce the risk of diseases in the community (Bezerra and Sichieri, 2011). A study on the relationship between food insecurity and diabetes in women found that they consume adequate diet, thus having a lower risk of diabetes than others (Seligman et al., 2007). Himalayan communities make good use of local herbs and spices that increase appetite and healing properties (Joshi et al., 2015). Allium species are used as spices and vegetables, and many of the species own strong medicinal purpose in the form of fresh paste or tonic (Keusgen et al., 2006). *Curcuma longa*, a major ingredient in the local kitchen, has miraculous effects on human ailments, thus being the consumer choice for cancer prevention, liver protection, treating wounds, and other activities in traditional medicine (Nita Chainani-Wu, 1982). Traditional food and recipes help address seasonal health issues, and diversity of recipes provide a supporting base for the nutritional security of the communities in the state of Uttarakhand (Mehta et al., 2010). Other than providing food security, the traditional food system also reduces the risk of deficiency in chronic health conditions such as obesity, high blood pressure, and higher cholesterol as well as nutrition increases the mental efficiency, physical development of children, etc. (Cole and Fox, 2004; Seligman et al., 2007; Borborah et al., 2014; Martin et al., 2016).

The health of women and children has been an enduring concern all over the state and the country (Anonymous 2014). As reported, the status of nutrition in Bageswar exhibited children influenced with stranded growth, underweight, and anemia. Also, the women of reproductive age were anemic. Uttarakhand comprised 25% of children with low birth weight (<2.5 kg) which is similar to the statistics in Bageshwar district (Pradhan 2017). A major reason for this is the lack of a health care delivery system to supply even the low-cost appropriate medical technology to all women and newborns. Moreover, the low purchasing power of the community for procuring tonics and related medicines is another reason. The state has been implementing *Janani Suraksha Yojana* (Women Safety Scheme) that provides financial assistance to mothers after delivery and *Janani–Shishu Suraksha Karyakram* (Mother–child safety scheme) for ensuring better facilities for women and child health services along with providing immunization, vitamin A, and iron supplement at childbirth (Anonymous 2012). The efficiency of such schemes needs to be improved in terms of ensuring universal access to all pregnant women and children, particularly in villages. Most of the dietary requirements are being met from traditional sources without which the magnitudes of malnutrition would have been much larger. Therefore, the role of traditional diets could not be undermined as, at least, it is helps maintain a certain dietary level, although it also necessitates strengthening the traditional dietary system to meet adequate nourishment requirements, particularly for lactating mothers and infants. The local community should be made aware of the nutritional role of local crops and plants for treating anemia, stunted growth, and lactation for which *Eleusine coracana*, *Setaria italica*, *Glycine max*, *Amaranthus caudatus*, *Fagopyrum esculentum*, *Vigna umbellata*, *Phaseolus vulgaris*, *Spinacia oleracea*, and *Chenopodium album* are greatly helpful. In addition, there are many traditional dishes that are supportive during such illnesses.

Despite clear distinguishing features and health benefits, increasing population, degradation of natural resources, biodiversity loss, soil degradation, and climate change, etc. are threatening to traditional agriculture systems. In addition, a change in cultural, social, and spiritual values has posed a significant threat to the traditional food system. A discussion with the community highlighted that the dependence on traditional food crops was more prevalent until the last decade. However, slowly, such practice is changing because of varied reasons. Traditional agriculture is labor-intensive and is practiced in small and fragmented landholdings. Because of its subsistence-type nature, farmers lack a surplus to sell in the market. This led to the switch on to other crops or the abandonment of agriculture fields. It creates a threat to a large variety of local crop gene pools. The young generation due to widespread education is looking for white-collar jobs, thus migrating to towns and cities. In addition, due to globalization and access to markets, food habit is changing. Some youths also feel that consuming traditional crops and food recipes is inferior in comparison to many other crops available in markets. All these factors influence traditional crops and food habits. Another possible reason for this is that diverse nutrient-rich local foods have gained limited consideration in the policy and decision-support process. Modern industrial agriculture and food systems have largely evolved through a centralized approach that brings in several hidden costs, including environmental as well as degradation of natural resources, etc. (Niketan et al., 2018). Such an approach has been a major threat to the biological and nutritional diversity of traditional diets (Fróna et al., 2019). High-cost market-driven and industrial crops are being promoted through a centralized approach. There is disconnect between agricultural and food policy and adequate nutrition supply (Pingali 2015). An analysis of India’s agricultural and food policy showed an inclination toward industrial food system. To address the issue of malnutrition and hunger and achieve Integrated Child Development targets, the country implemented the public distribution system (PDS) and mid-day meal scheme; however, they are more market-driven and frequently complain about micronutrient deficiencies in many areas. Inconsiderately, the state agencies also promote a market-based agricultural system. There is a need to make traditional agriculture and farming systems more reliable and sustainable (FAO 2017). It would be highly disadvantageous if the community lost such an efficient system or changes their agroecological practices. This highlights a need to have a realistic way forward to protect native food systems and diets so that they can continuously support food, nutrition, and health benefits.

### Way Forward

The implications of this research are of both academic importance and practical significance to ensure food–medicine security and avoid malnutrition among rural communities. For food security, a sustainable food production system is highly demanding. The study established a better understanding of the intersection between traditional food, dietary diversity, nutritional quality, and medicinal efficacy. It highlighted the need to conserve and continue with the traditional food systems as it is in the best interest of the people. We expect that the study would receive greater attention from policy planners and developmental workers that will not only salvage the dwindling traditional dietary system but also ensure a healthy dietary cover to the marginal hill communities. The local government should recognize the role of traditional food and dietary systems in meeting community necessities and support appropriate policies and programs to continue with it. It should support better financial incentives to farmers to continue traditional agriculture and dietary practices. It would empower the local communities to manage traditional food and dietary systems not only to feed themselves and preserve crop gene pools but also to sell produces in markets. Greater awareness of the masses and policy planners is desired on the beneficial aspects of traditional crops and wild plants. In addition, proper documentation of traditional knowledge related to local crops, cropping practices, local delicacies and recipes, and herbal preparation is highly desirable for future use. Such an approach will not only promote and nurture traditional food systems but also support local sociocultural and ecological systems and provide a sustainable solution for nutrition security. This requires location-specific strategies and trade-offs so as to develop a synergy in terms of food security and environmental gains with minimum transformations. Other than government support, public investment in various forms is also required. In addition, there is a need for proper scientific investigation on nutritional/anti-nutritional and therapeutic efficiencies in food items and herbal preparation as the current state of knowledge is highly limiting on these aspects. There is a need to make some traditional food, recipes, cuisines, and ingredients appropriate for recent times that may attract a significant population outside. The local government should incentivize the traditional system so that it creates new values and job opportunities for youngsters. It is expected that the study would lead to advancement of a renewed thinking on traditional agriculture for food and nutritional security as well as sustainable rural development all over. It is a global challenge to conserve traditional food systems and maintain healthy diets that have been sustainably used for generations and can have an important synergy with SDGs (Wang et al., 2018).

## Data Availability

The original contributions presented in the study are included in the article/Supplementary Material; further inquiries can be directed to the corresponding author.
