# Supplementary material for: Traditional Dietary Knowledge of a Marginal Hill Community in the Central Himalaya: Implications for Food, Nutrition, and Medicinal Security
Source: Front Pharmacol. 2022 Mar 30;12:789360. doi: 10.3389/fphar.2021.789360 (PMC9006986; doi:10.3389/fphar.2021.789360)
Supplement: Supplementary file 1 [file DataSheet1.pdf]

Annexe 1. Nutritional status of crop plants used as traditional foods and medicinal plants in the Central Himalaya

Crop Species	Plant part used	Nutritional parameters (per 100 gm)													
		Moisture (%)	Energy (KCal)	Protein (g)	Fat (g)	CHO (g)	Vit. B1 (mg)	Vit. C (mg)	Vit. D (µg)	Vit. E (mg)	Ca (mg)	Fe (mg)	Mg (mg)	Na (mg)	Zn (mg)
Allium cepa L.	B	88.35	26.76	2.07	0.26	20.99	0.03	27.33	6.81	-	31.1	3.09	66.7	15.52	0.45
*Allium sativum*L.	B	64.42	122.84	6.75	0.14	21.84	0.2	13.57	1.97	0.06	17.6	0.88	25.78	10.56	0.81
[2]*Allium schoenoprasum* L.	C&L	90.65	30.00	3.27	0.73	4.35	0.07	58.1	0.00	0.21	92.0	1.60	42.00	3.00	0.56
Amaranthus caudatus L.	S	9.2	355.87	13.27	5.56	61.46	0.04	0	53.98	0.15	162	8.03	270.0	2.81	2.52
[2]*Asparagus* spp	Ts	93.22	22.00	2.2	0.12	3.88	0.14	5.6	-	1.13	24.0	2.14	14.00	2.00	0.54
[4]*Bauhinia variegata* L.	Fb	73.95	385.98	4.49	2.42	86.56	-	4.39	-	-	6.15	0.06	0.06	0.48	0.04
Benincasa hispida Thunb.	Fr	92.17	73.00	0.79	0.14	2.84	0.03	11.4	1.35	0.02	19.3	0.47	19.95	0.77	0.13
Brassica campestris L.	-	-	-	-	-	-	-	-	-	-	-	-	-	-	-
B. juncea(L.) Czern	So	5.67	509.54	19.51	40.1	16.8	0.55	0	31.79	8.2	191	2.84	51.63	19.41	0.68
[5]*Brassica nigra* (L.) Koch	S	4.16	-	2.47	3.03	3.54	-	-	-	-	-	-	-	-	-
Brassica oleracea var. *capitata*	Vb	91.85	90.00	1.36	0.12	3.25	0.04	33.25	0.21	0.05	51.7	0.35	17.99	14.98	0.16
[2]*Cannabis sativa* L.	S	4.96	553.00	31.56	48.7	8.67	1.28	0.50	-	0.80	70.0	7.95	700.0	5.00	9.90
Capsicum annuum L.	Fc	93.89	16.25	1.11	0.34	0.84	0.05	123.78	0.7	0.07	14.7	0.48	11.84	1.84	0.15
Chenopodium album L	Lt	88.77	3.82	2.5	0.44	2.56	0.06	41.03	1.01	0.25	211	2.66	48.41	10.75	0.98
Citrus hystrix DC.	Fr	-	-	-	-	-	-	-	-	-	-	-	-	-	-
Citrus limon (L.) Burm. f.	Fr	91.59	153.00	0.41	0.75	6.97	0.04	48.16	0.39	0.06	22.6	0.12	8.9	1.21	0.08
Colocasia esculenta L.(Corm)	Co	73.49	88.91	3.31	0.17	17.85	0.06	1.83	0.27	0.33	30.1	0.66	36.93	4.54	11
Colocasia esculenta L. (	Pe	92.67	19.36	0.91	0.22	2.86	0.02	5.83	0.14	0.09	40.2	0.55	19.56	0.60	20
Coriandrum sativum L.	S	86.99	31.07	3.52	0.7	1.93	0.09	23.87	3.55	0.46	146.	5.3	72.68	37.00	0.68
Cucumis sativus L.	Fr	92.96	19.59	0.71	0.16	3.48	0.02	6.11	1.26	0.02	16.3	0.46	20.38	6.33	0.17
Cucurbita moschata Duch. ex	Fr	91.85	23.18	0.84	0.16	4.00	0.03	8.04	1.40	0.60	23.0	0.36	10.43	8.81	0.11
*Curcuma longa*L.	Rh	10.58	280.58	7.66	5.03	49.22	0.06	13.86	18.67	2.92	122.	46.0	260	24.41	2.64
[6]*Cyclanthera pedata* (L.) Schrad	Fr	94.00	17.00	0.60	0.10	4.00	0.04	14.00	-	-	14.0	0.80	-	-	-
Dioscorea alata L.	Tu	69.6	118.00	1.53	0.17	27.88	0.11	17.1	-	0.35	17.0	0.54	21.00	9.00	0.24
Dioscorea bulbifera L.	AT	9.78	--	5.86	--	73.87	0.42	0.63	--	--	378.	3.14	128.7	87.80	2.79
[7]*Diplazium esculentum*(Retz.) Sw.	Fro	-	-	1.08	-	52.14	-	-	-	-	15.1	10.3 9	19.2	17.2	1.58
[8]*Echinochloa frumentacea* Link.	S	2.50	398.00	2.87	2.60	80.93	0.33	-	-	-	2.74	0.05	4.32	0.83	-

Eleusine coracana (L.) Gaert.	S	10.89	320.73	7.16	1.92	66.82	0.37	-	41.46	0.16	364	4.62	146	4.75	2.53
[9]*Fagopyrum esculentum*	S	11.0	335.00	12.00	7.40	72.9	3.30	-	-	40.00	110.	4.0	390.0	-	0.8
[10]*Fagopyrum cymosum* (Trev.) Meisn	L	-	-	13.10	1.89	-	0.47	-	-	-	31.00	87.00	212.00	41.00	1.78
[1]*Ficus palmata* Forssk.	Fr	48.20	107.37	4.06	-	20.78	-	0.83	-	-	1.54	0.18	-	-	-
[1]*Ficus auriculata* Lour.	Fr	87.91	-	-	-	-	-	5.48	-	-	15.6	5.43	68.00	29.00	-
Glycine max (L.) Merrill	S	5.51	381.44	35.58	19.8	12.79	0.59	-	66.22	1.29	240.	8.29	259.0	2.07	4.01
Glycine max subsp. *soja* (Sieb. & Zucc.) H. Ohashi	S	8.52	451.00	43.62	19.25	25.89	-	-	-	-	268.00	9.84	264.54	-	4.79
Hordeum vulgare L.	S	9.77	315.71	10.94	1.3	61.29	0.36	-	-	0.01	28.6	1.56	48.97	7.56	1.5
Lagenaria siceraria (Molina)	Fr	95.17	10.99	0.53	0.13	1.68	0.03	4.33	0.74	0.02	15.4	0.26	10.93	1.46	0.15
Lens culinaris Medik	S	9.71	322.41	24.35	0.75	52.53	0.34	-	1.31	0.16	44.3	7.06	74.69	10.27	3.61
Lepidium sativum L. (Seeds)	S	4.6	445.25	23.36	23.7	33.66	0.52	-	1.92	0.07	217	6.19	79.24	25.35	1.52
Lepidium sativum L. (Leaf)	L	84.02	49.71	5.62	0.80	4.48	0.33	42.75	0.55	0.74	318	17.2	307	21.84	4.83
Linum usitatissimum L.	S	5.48	443.82	18.55	35.6	10.99	0.28	-	0.55	8.28	257.	5.44	349.0	32.93	4.86
Luffa acutangula (L.) Roxb.	Fr	94.99	13.14	0.91	0.14	1.72	0.02	5.42	0.37	0.02	13.7	0.42	16.15	4.71	0.22
Macrotyloma uniflorum (Lam.) Verde	S	9.28	329.58	21.73	0.62	57.24	0.32	-	1.8	0.27	269	8.76	152	12.14	2.71
Megacarpaea polyandra Benth.	L	-	-	-	-	-	-	-	-	-	-	-	-	-	-
Mentha longifolia L.	L	84.24	37.04	4.66	0.65	2.39	0.02	17.16	3.37	0.46	205	8.56	110	16.87	0.75
Momordica charantia L.	Fr	91.60	79.00	1.34	0.24	2.53	0.06	50.87	1.90	0.03	16.2	1.08	31.58	12.59	0.36
[2]*Ocimum basilicum* L.	L	-	23.00	3.15	0.65	2.65	0.03	18.0	-	0.80	1.77	3.17	64	4	-
*Oryza sativa*L.	S	9.93	356.34	7.94	0.52	78.24	0.05	-	-	0.06	7.49	0.65	19.3	2.34	1.21
Phaseolus vulgaris L.	S	9.87	299.23	19.91	1.77	48.61	0.30	-	26.73	0.23	126.	6.13	173.0	10.43	2.69
[2]*Phytolacca acinosa* Roxb.	L	91.6	23.00	2.60	0.40	3.70	0.08	136.00	-	-	53.0	1.70	18.00	23.00	0.24
Punica granatum L. (fruit)	Fr	83.55	54.73	1.33	0.15	11.58	0.06	12.69	109.00	0.03	10.65	0.31	11.07	2.13	0.18
Psidium guajava L.	Fr	83.79	32.26	1.44	0.32	5.13	0.05	214.00	1.68	0.09	18.5	0.32	15.26	2.87	0.23
Raphanus sativus L.(Leaves)	WP	91.19	26.05	2.22	0.51	2.77	0.06	65.76	1.39	0.08	234	3.82	57.96	17.39	0.49
[3]*Ricinus communis* L.	S	7.18	-	22.11	-	-	-	-	-	-	19.3	17.0	49.95	74.32	-
Sesamum indicum L.	S	4.51	507.63	19.17	43.1	10.29	0.34	-	67.83	0.09	1664	13.9	390	15.91	8.59
Setaria italica(L.)P. Beauv	S	14.23	331.73	8.29	2.55	66.19	0.29	-	-	0.07	15.2	2.34	122	3.35	1.65
Solanum melongena L.	Fr	90	25.33	1.48	0.32	3.52	0.06	2.09	1.04	0.07	16.5	0.37	21.5	3.55	0.21
Solanum tuberosum L.	St	80.72	69.79	1.54	0.23	14.89	0.06	23.15	0.19	0.06	9.52	0.57	24.07	4.11	0.28
Spinacia oleracea L.	L	90.31	24.38	2.14	0.64	2.05	0.16	30.28	0.26	1.29	82.2	2.95	86.97	42.55	0.46

Syzygium cumini (L.) Skeels	Fr	83.33	56.16	0.82	0.17	12.3	0.02	16.47	0.82	0.04	25.3	0.33	334	183	1.13
Trachyspermum ammi (L.)Spr.	S	9.71	357.30	15.89	21.1	24.53	0.3	51.79	2.62	0.01	1034	13.6	273.2	28.58	5.67
Trichosanthes anguina L.	Fr	94.81	12.42	0.98	0.25	1.27	0.03	2.72	2.67	0.01	24.6	0.32	18.7	7.07	0.14
Trigonella foenum–graecum L.	L	86.7	34.41	3.68	0.83	2.17	0.11	58.25	2.36	0.36	274.	5.69	63.67	47.01	0.54
Trigonella foenum–graecum L.	S	7.82	234.93	25.41	5.72	10.57	0.28	-	1.98	1.5	135.	8.47	167	40.2	3.8
Triticum aestivum L.	S	11.1	320.26	10.57	1.53	64.17	0.42	-	13.43	0.26	30.9	4.10	125.0	2.04	2.85
[2]*Urtica ardens* Link.	L	87.00	42.00	2.71	0.11	7.49	0.008	-	-	-	481.	1.64	57.00	4.00	0.34
Vigna mungo (L.) Hepper	S	9.16	324.08	23.06	1.69	51.00	0.21	-	8.42	0.17	55.6	4.67	173.0	18.88	3.00
Vigna umbellata (Thunb.) Ohwi& Ohashi	S	11.12	302.33	19.97	3.54	51.26	0.46	1.11	8.26	21.85	200.00	4.76	201.00	10.62	2.29
Vigna unguiculata (L.) Walp	S	9.32	320.26	21.25	1.14	53.77	0.34		0.93	0.65	81.7	5.04	213.0	12.52	3.57
Zea mays L. (Dry)	S	9.26	334.12	8.80	3.77	64.77	0.33	-	33.6	0.36	8.91	2.49	145	4.44	2.27
Zingiber officinale Roscoe	Rh	81.27	54.97	2.22	0.85	8.97	0.04	5.43	4.09	0.32	18.8	1.9	54.66	10.03	0.39

Source: All valued are based on ICMR-ICFT (2017) except for[1]Saklani & Chandra, 2011 & 2012; [2]USDA National nutrient data base; [3]Annongu & Joseph, 2008, Akande et al., 2012; [4]Verma et al., 2012; [5]Uzama et al., 2016; [6]Dhyani & Dhyani, 2014; [7]Tewari et al, 2020; [8]Chandra et al., 2018; [9]Joshi et al., 2015; [10]Zhao *et al.*, 2004.

Plant part used: Bulb (B); Cloves & Leaf (C&L); Seed (S); Tender shoots (Ts); Flower buds (Fb); Fruit (Fr); Seed oil (So); Vegetative buds (Vb); Fruit capsule (Fc); Leaf twig (Lt); Corms (Co); Petiole (Pe); Rhizome (Rh); Tuber (Tu); Aerial tuber (ATu); Fronds (Fro); Whole Plant (WP); Stem (St); Leaf Twig (LTw)

Annexe 2. Reported major chemical compounds and medicinal importanceof the traditional crops in the Central Himalaya

Species	Major Chemical Compounds	Reported Uses	Source
Allium cepa L.	Allyl propyl disulfide; Cysteine sulphoxide; Catechol; Di-allyl methyl; Methyl allyl; Propanethiol; Phloroglucinol; Thio propiono aldehyde; Thiocynate etc.	Aphrodisiac; Anti-cancer; Analgesic; Anti-anaemic; Anti-microbial (fungal & bacterial); Anti-diabetic; Cardio-protective; Earache; Hepatoprotective; Reduce swelling & cholesterol; Lower blood sugar levels; Remove warts etc.	Borborah et al., 2014.
Allium sativum L.	Agoene; Allicin; Alliin; Allistatin; Allyl propyl disulfide; Cysteine;Glutamine; Isoleucine, etc.	Alzheimer; Anti-microbial (fungal, bacterial & viral); Anti-cancer; Anti-diabetic; Anti-tumour; Diuretic; Digestive; Cardio-protective; Hepatoprotective; Immuno-modulatory; Radioprotective; Wound healing etc.	Borborah et al., 2014; Alam et al., 2016.
Allium schoenoprasum L.	Alliin; S-allylcysteine; S-methylcysteine; S-ethylcysteine.	Anti-cancer; Anti-diabetic activity; Anti-microbial (fungal & bacterial); Anti-oxidative; Cytotoxic & anti-tumour; Cardio protective; Digestive; Hepatoprotective effect, Immunomodulatory Potential; Wound healing.	Borborah et al., 2014; Parvu et al., 2014
Amaranthus caudatus L.	Alkaloids; Flavonoids; Lycosides; Saponins; Tanins; Triterpenoids etc.	Anti-helmenthic; Anti-hyperglycaemic; Anti-microbial (fungal & viral); Anti-nociceptive; Anti-oxidant; Hepatoprotective.	Reyad-ul-Ferdous, 2015.
Asparagus filicinus Buch.-Ham. ex D.Don	Aspafilioside A, B &C;); 26-diol (Filicinoside A); 26 triol (Filicinoside B); Furostanoside; Officinalisnin II; Oligo-furostanosides (Filicinis A&B); Oligo-spirostanosides (Filicinoside C&D); Tormentic acid.	Anti-oxidant; Antipyretic; Anti-tussive; Diuretic; Expectorant; Menstrual problem; Nervous stimulant Stomachic; Tonic.	Mishra et al., 2017; Negi et al., 2010
Bauhinia variegata L.	α-alanine; α-ketoglutaric acid; Aspartic acid; Glycine; Glutamic acid; Oxaloacetic acid; Phosphoenolpyruvic acid; Serine.	Anti-goitrogenic; Anti-inflammatory; Anti-Obesity; Anti-arthritic; Anti-ulcer; Hepatoprotective; Immunomodulatory;	Bansal, et al., 2014; Shahana and Nikalje, 2017.
Benincasa hispida Thunb.	Flavone (Iso-vitexin); Sterols (Lupeol, Lupeol acetate) Triterpenes (Alnusenol, Isomultiflorenol, Multiflorenol); and β-sitosterol.	Anti-angiogenic; Anti-depressant; Anti-diarrheal; Anti-oxidant; Anti-ulcer; Blood diseases; Dyspepsia; Epilepsy; Gastroprotective; Jaundice; Menstrual disorders.	Pagare et al., 2011.
Brassica campestris L.	Hydroxycinnamic acids; Isothiocyanate; Gluconapin; Isorhamnetin; Kaempferol; Linoleic acid; Linolenic acid; Malic acid, Oleic acid; Palmitic acid; Quinic acid; Quercetin; Sinapic acids etc.	Analgesic; Anthelmintic; Anti-cancer; Anti-diabetic; Anti-microbial (fungal & bacterial); Anti-inflammatory; Anti-oxidant; Anti-obesity; Aphrodisiac;	Kumar et al., 2011; Kumar & Andy, 2012; Negi et al., 2013; Rahman et al., 2018.

		Cardiovascular;Diuretic; Hepatoprotective; Hypolipidemic etc.	
Brassica juncea(L.) Czern	Allyl Isothiocyanate; Caffeic acid; Gallic acid; Ferulic acid; Methallyl cyanide; p-coumaric acid; p-hydroxy benzoic acid; Phytic acid; Sinapic acid etc.	Anti-allergenic; Anti-cardiovascular; Anti-carcinogenic; Anti-diarrheal; Anti-inflammatory; Anti-microbial (fungal & bacterial); Anti-oxidant; Anti-ulcer etc.	Mehta et al., 2010; Negi et al., 2013; Sharma & Rai, 2018.
Brassica nigra (L.) Koch	Alkaloids; Flavonoids; Glycosides; Phlobatannins; Saponins; and volatile oil.	Anti-cancer; Anti-catarrhal; Anti-diabetic; Anti-microbial (bacterial); Anti-spasmodic; Appetizing; Arthritis; Cold; Diuretic; Emetic; Flu; Laxative; Rheumatism and Stimulant.	Shankar et al., 2019; Uzama et al., 2016.
Brassica oleracea var. *capitata* L.	Amino acids; Carbohydrates; Flavonoids; Proteins; Phenolic compounds and Tannins.	Anti-oxidant; Anti-inflammatory and Anti-microbial (bacterial).	Chauhan & Singh, 2019; Shankar et al., 2019.
Cannabis sativa L.	Amino acids; Cannabinoids (C21 terpenophenolic); Flavonoids; Hydrocarbons; Nitrogenous compounds; Steroids; Terpenes.	Anti-cancer; Anti-diabetic; Anti-diuretic; Anti-depressant; Anti-inflammatory; Anti-microbial; Anti-nausea; Anti-vomiting; Anti-spasmodic; Anthelmintic; Aphrodisiac; Hyperglycemic; Immunomodulatory etc.	Kuddus et al., 2013
Capsicum annuum L.	Amino acids; Capsaicin; Caffeic acid; Capsicoside A-D; Carvone, Chlorogenic acid; Cinnamic, Citric acid; Furostanol; Solanine; Solanidine; Zeaxanthin; Vitamin B1,B3,C,E etc.	Analgesic; Anti-angiogenic; Anti-diabetic; Anti-obesity; Anti-microbial (fungal, bacterial & viral); Anti-parasitics; Anti-spasmodic; Cardiovascular; Gastro-protective; Immuno-suppressants; Larvicidal; Stomachic, Stimulant.	Fathima, 2015; Sanati et al., 2018.
Chenopodium album L	Alkaloids; Flavonoids (Kaempferol, Quercetin); Lysine; Phytosterols (B-Sitosterol, Lupeol); Saponins (Cinnamic acid amides) etc.	Anthelmintic; Anti-pruritic; Anti-nociceptive; Anti-hypolipidemic; Antioxidant; Cardiotonic; Diuretic and laxative; Sperm immobilization etc.	Poonia & Upadhayay, 2015.
Citrus hystrix DC.	Carotenoids; Coumarins; Flavonoids; Psoralen, etc.	Analgesic; Anti-allergic; Anti-anxiety; Anti-carcinogenic; anti-depressant; Anti-inflammatory; Anti-microbial; Appetizer; Cardiac stimulant etc.	Sidana et al., 2013.
Citrus limon (L.) Burm. f.	Geraniol; Limonene; Linalool; Linalyl acetate; Methyl 2-methyl-2-butenyl ether; α-Citral (geranial); α-Pinene; α-terpineol; β-Citral (neral); β-Pinene; γ-Terpinene.	Anti-inflammatory; Anti-microbial (bacterial); Anti-cancer; and Anti-parasitic.	Klimek-Szczykutowicz et al., 2020; Semwal et al., 2015.
Colocasia esculenta L.	Anthocyanins; Calcium oxalate; Flavonoids; Globulins; Ttriterpenoids; Vitamin A, B, C; β-sitosterol; Steroids etc.	Analgesic; Anti-cancer; Anti-diabetic; Anti-helminthic; Anti-microbial (bacterial & fungal); Anti-inflammatory.	Pawar et al., 2018.

Coriandrum sativum L.	Camphor; Camphene; Coriandrin; ρ-cymene; Geraniol; Linalol; Myrcene; Limonene; α- & β-pinene; α-terpineol; γ-terpinene etc.	Analgesic; Anti-diabetic; Anti-diuretic; Anti-inflammatory; Anti-microbial (fungal, bacterial & viral); Anti-oxidant; Anti-spasmodic; Anti-rheumatic; Anxiolytic; Carminative; Digestive; Hepatoprotective etc.	Ramadan, & Morsel, 2002; Mahendra & Bisht, 2011; Asgarpanah, 2012.
Cucumis sativus L.	Apigenin; Cucubitacins; Cucumerin; Isocoparin; Orientin; Vitexin etc.	Anti-diabetic; Anti-inflamatory; Anti-tumor; Artherosclerosis etc.	Mallik et al., 2013.
Cucurbita moschata Duch. ex Poir.	Carotenoids; Essential amino acids; Flavonoids; Phenolics; Polysaccharides; Proteins; Vitamins (βcarotene, Vitamin A, B2, α-tocopherol, C, E) etc.	Anti-diabetic; Anti-hypertension; Anti-inflammation; Anti-microbial (bacterial & fungal); Anti-oxidant; Anti-tumor; Anti-ulcer; Iimmunomodulation;	Suresh & Sisodia, 2018.
Curcuma longa L.	Caryophyllene; Curcumin; Curzerenone; Curdione; p-cymene; Geraniol; Mono-& di-demethoxy curcumin; Linalool; α-& β-pinene; Myrcene; α-phellandrene; 1,8-cineole, Sabinene; Tumerone a & b etc.	Anti-allergic; Anti-cancer; Anti-coagulant; Anti-diabetic; Anti-fertility; Anti- inflammatory; Anti-microbial (fungal & bacterial); Anti-oxidant; Anti-ulcer; Anti-venom; Digestive; Hepatoprotective; Immunity booster; Nephroprotective etc.	Nasri et al., 2014.
Cyclanthera pedata (L.) Schrad	Anthocyanin; Ascorbic acid; Polyphenolic compounds; Flavones; Flavonols; Tannins etc.	Anti-inflammatory; Anti-oxidant; Hypoglycemic; Hypocholesterolemic etc.	Rivas et al., 2013.
Dioscorea alata L.	Diosgenin; Glycosides; Flavonoids; Sapogenin; Smilagenin; β-isomer yammogenin; Phenolic compounds etc.	Anti-diabetic; Bone disease; Cardiovascular; Central nervous system disorders; Disgestive disorders; Metabolic disorder; Skin diseases, Immune deficiency and autoimmune diseases; Neuroprotective etc.	Mustafa et al., 2018.
Dioscorea bulbifera L.	Cardio-glaconoids; Diosgenin; Flavonoids; Saponin; Terpenoids etc.	Anthelmintic; Anti-asthmatic; Anti-cancer, Anti-diabetic; Anti-hyperglycaemic; Anti-inflammatory; Anti-microbial (fungal & bacterial); Anti-parasitic; Anti-tumour; Diuretic; Gastro-regulatory; Rejuvenating etc.	Ghosh, 2015; Ezeabara & Regina, 2018.
Diplazium esculentum(Retz.) Sw.	Alkaloids; Anthraquinones; Di-terpenes; Flavonoids; Gum & Mucillages; Phenols; Saponins; Steroids; Tannins,; Tri-terpenes etc.	Anti-biotic; Anti-inflammatory; Anti-oxidant; Anthelmintic; Anti-microbial; Cytotoxic; Larvicidal activity etc.	Tongco et al., 2014;
*Echinochloa frumentacea*Link.	Alkaloids; Carbohydrates; Glycosides,; Flavonoids; Resins; Saponins; Tannins; Terpenoids.	Anti-inflammatory; Anti-diabetic; Anti-oxidant; Anti-cancer; Cardiovascular; Phytoestrogen etc.	Chandra et al., 2018.
Eleusine coracana (L.) Gaert.	Anthocyanins; Flavan-4-als; Phenols: Tanins; Tocoferols etc.	Anti-diabetic; Anti-constipation; Anti-inflammatory; Anti-microbial; Cardiovascular disease etc.	Devi et al., 2014.

Fagopyrum esculentum Moench	Amino acid (Methionine); Flavonoids (Rutin, Quercetin, Orientin, Homoorientin, Vitexin & Isovitexin); Phenolic compounds etc.	Anti-diabetic; Anti-oxidant; Anti-inflammatory; Anti-carcinogenic; Anti-tumor; Cholesterol reducing; Hypertensive; Hyperglycemic; Hyperlipidemia.	Joshi et al., 2019.
Fagopyrum cymosum (Trev.) Meisn	Amino acid (Methionine); Flavonoids (Rutin, Quercetin, Orientin, Homoorientin, Vitexin & Isovitexin); Phenolic compounds etc.	Anti-diabetic; Anti-oxidant; Anti-inflammatory; Anti-carcinogenic; Anti-tumor; Cholesterol reducing; Hypertensive; Hyperglycemic; Hyperlipidemia.	Joshi et al., 2015.
Ficus palmata Forssk.	Alkaloids; Bergapten; Cardian glycosides; Coumarins; Flavonoids; Psoralenoside; Sitosterol; Tannins; Terpenoids; Triterpene; Vannilic acid etc.	Anti-calcinogenic; Anti-coagulant; Anti-microbial; Anti-oxidant; Anti-proliferative; Anti-ulcer; Hepatoprotective; Nephroprotective etc.	Joshi et al., 2014.
Ficus auriculata Lour.	Alkaloids; Coumarins; Maslinic acid; Flavonoids; Protocatechuic acid; Saponins; Steroids;Tannins; Triterpene (Oleanolic acid) etc.	Anti-inflammatory; Anti-oxidant; Anti-ulcer; Gastro protective; Hepatoprotective; Radioprotective etc.	Sirisha et al., 2010.
Glycine max (L.) Merrill	Alpha-linolenic acid; Daidzein; Genistein; Isoflavones; Phenolic acids; Saponins etc.	Anti-arthritic; Anti-cancer; Anti-diabetic; Anti-hypersensitive; Anti-inflammatory; Anti-oxidative; Anti-nociceptive; Anti-obesitive; Anti-schemic; Anti-viral; Cardio protective; Hepatoprotective etc.	Kanchana et al., 2016.
Glycine max subsp. *soja* (Sieb. & Zucc.) H. Ohashi	Alkaloids; Anthocyanins; Flavonoids; Glycosides; Saponins; Sterols; Phenolics; Phytic acid; Saponins; Tri-terpenoids, Tanins etc.	Anti-arthritic; Anti-cancer; Anti-cataract; Anti-carcinogenic; Anti- diabetic; Anti-hyperlipidemic; Anti-infertility; Anti-obesity; Anti-oxidant; Anti-inflammatory; Nephroprotective; Wound healing etc.	Hidayat and Dwira, 2018; Ganesan & Xu, 2017.
Hordeum vulgare L.	Glutathione; Phenolic acids; Phytic acids; Sphingolipids; Tocotrienols etc.	Anti-carcinogenic; Anti-diabetic; Anti-inflammatory; Anti-obesity etc.	Gul et al., 2014.
Lagenaria siceraria (Molina) Standl.	Alkaloids; Carotene; Cucurbitacin; Flavonoids; Lagenin; Polyphenols; Saponins; Triterpenoids etc.	Anti-bilious; Anti-helmintic; Anti-inflamatory; Analgesic; Antidote; Cardio protective; Diuretic; etc.	Prajapati et al., 2010; Ahmad et al., 2011.
Lens culinaris Medik	Carotenoids; Phenolic acids; Phytic acids; Phytosterol; Saponins; Tanins; Tocopherols etc.	Anti-inflammatory; Anti-oxidant etc.	Zhang et al., 2018.
Lepidium sativum L. (Seeds)	Ascorbic acid; Carotenes; Imidazole; Lepidine; Oleic acid; Palmitic acid; Sinapin; Stearic acid etc.	Anti-diabetic; Anti-diarrheal; Anti-hypersensitive; Anti-inflammatory; Anti-microbial; Anti-spasmodic; Brochio-asthma; Chemoprotective; Diuretic; Hypoglycemic; Hepatoprotective; Laxative etc.	Shail et al., 2016.

Linum usitatissimum L.	Alkaloids; Coumarins; Flavonoids; Glycosides; Quinines; Phenols; Saponins; Steroids; Tanins; Terpenoids, etc.	Anti-allergic; Anti-arrhythimic; Anti-carcinogenic; Anti-hypersensitive; Anti-microbial (fungal & viral); Anti-oxidant,; Anti-parasitic; Anti-spasmodic Immuno-stimulant etc.	Monica & Joseph, 2016; Mohamed El-Feky et al., 2016.
Luffa acutangula (L.) Roxb.	Arginine; Alanine; Carotene; Cysteine; Glycine; Hydroxyprolines; Leucine; Luffeine; Phytin; Serine; Saponins; Tryptophan etc.	Anti-cholinegenic; Anti-hypersensitive; Anti-inflammatory; Anti-parasitic; Anti-ulcer etc.	Anitha & Miruthula, 2014.
Macrotyloma uniflorum (Lam.) Verde	Cinnamic acids; Isoflavones; Lignans; Phytic acids; Phenolic acids; Phytates; Saponins; Tannins etc.	Anti-carcinogenic; Anti-inflammatory; Anti-oxidative; Astringent; Anti-ulcer; Gastro-protective etc.	Prasad & Singh, 2015.
Megacarpaea polyandra Benth.	--	--	--
Mentha arvensis L.	Carvone; Cis-piperitone; 1,8-cineole; Cis-carvyl acetate; Dihydrocarveol; Germacrene D; Isomenthone; Menthone; Piperitenone; Pulegone etc.	Analgesic; Anti-catarrhal; Anti-emetic; Anti-inflammatory; Anti-microbial; Antispasmodic; Anti-oxidative; Anti-pyretic; Carminative; Gastro-protective; Stimulant etc.	Joshi et al., 2016; Okut et al., 2017.
Momordica charantia L.	Alkaloids; Anthraquinones; Anthocyanins; Coumarins; Emodins; Flavonoids; Glycosides; Saponins; Terpenoids; etc.	Anti-anaemic; Anti-diaabetic; Anti-leukemic; Anti-microbial (viral); Anti-oxidant; Anti-ulcerative; Contraceptive; Immunoregulatory etc.	Adi & Reddy., 2017; Daniel et al., 2014.
Ocimum basilicum L.	Ascorbic acid; 1,8-cineole; Cadinol; Eugenol; Geraniol; Linalyl acetate; Linalool; Methyl eugenol; Methyl chavicol etc.	Anti-microbial (bacterial, fungal & viral); Anti-diabetic; Anti-inflammatory; Anti-oxidant; Anti-spasmodic; Anti-toxic; Anthelmintic; Carminative; Hepatoprotective; Hypolipidemic; Immuno-modulatory; Stomachic etc.	Anand et al., 2011; Joshi et al., 2016.
Oryza sativa L.	Anthocyananins; Arabinoxylans; Flavonoids; Phenolic acids; Proanthocyanins; Riboflavin; Tocopherols; Tocotrienols, etc.	Anti-cancer; Anti-diabetic & controlling metabolic syndrome; Anti-inflammatory; Anti-oxidant; Dermatological uses; Immune-stimulant etc.	Burlando & Cornara, 2014; Jamil & Anwar, 2016.
Perilla frutescens (L.) Britton	Amino acids; Caffeic acid; Cinnamic acid; Flavonoids; Phenolic acids; Gallic acid; Polysaccharides; Protein; Rosmarinic acids etc.	Anti-Allergic; Anti-depressant; Anti-inflammatory; Anti-oxidant; Anti-cancer; Anti-cataract; Anti-tumor; Arteriosclerosis etc.	Ahamad, 2019
Phaseolus vulgaris L.	Alkaloids; Anthraquinones; Catechic tannins; Flavonoids, Gallic acids; Glycosides; Polyphenols; Saponins; Steroids; Tannins; Terpenoids.	Anti-carcinogenic; Anti-diabetic; Anti-oxidant; Anti-hyperglycaemic etc.	Ocho-Anin atchibri et al., 2010.

Phytolacca acinosa Roxb.	Acinosolic acid; Jailigonic acid; Lectins, Oleanolic acid; Myricadol; Phytolaccagenin; Spergulagenic acid; zonarol.	Anti- inflammatory; Anti-microbial; Anti-fungal; Purgative; Narcotic etc.	Kunwar et al., 2010.
Punica granatum L.	Ascorbic acid; Coumaric acids; Ferulic acids; Isosanoic acids; Linolenic acids; Stearic acids etc.	Anti-anaemic; Anti-carcinogenic, anti-diabetic; Anti-microbial (bacterial & fungal) etc.	Bhowmik et al., 2013.
Psidium guajava L.	Arabopyranoside; Ascorbic acid; Citric acid; Caryophyllene oxide; Guajadial; Methanolic extracts etc.	Anti-cancer; Anti-diarrheal; Anti-inflammatory; Anti-microbial (fungal); Anti-mutagenic; Anti-oxidative etc.	Naseer et al., 2018.
*Raphanus sativus*L.(Leaves)	Iisothiocyanate; Levons; Lysines; Methins; Methionine; Nicotinic acid; Raphanin; Riboflavin; Sapogenins; Sulphoraphene; Thiamine etc.	Anti-aging; Anti-carcinogenic; Anti-diarrheal; Anti-hypertensive; Anti-microbial (bacterial); Anti-oxidative; Anti-tussive; Constipation; Neuroprotective; Radioprotective etc.	Singh and Singh, 2013.
Ricinus communis L.	Alkaloids; Flavonoids; Phenols (Kaempferol, Ricin, Rutin, Thujone etc.); Saponins; Terpenes etc.	Analgesic; Anti-asthmatic; Anti-cancer; Anti-dermatophytics; Anti-diabetic; Anti-microbial (bacterial & fungal); Anti-nociceptive; Anti-oxidant; Anti-ulcer; Laxative; Insecticidal etc.	Abdul et al., 2018.
Sesamum indicum L.	Argenine; Cephalin; Histidine; Isoleucine; Methionine; Myristic acid; Phytosterol; Sesamin; Sesamolin; Tryptophan; Valine;, etc.	Analgesic; Anti-colic; Anti-dandruff; Anti-hypertensive; Anti-microbial (bacterial, fungal & viral); Anti-oxidant; Antidot; Aphrodisiac; Diuretic; Haemostatic etc.	Anila et al., 2010; Raghavan et al., 2010.
Setaria italica (L.) P. Beauv.	Alkanoids; Flavanoids; Saponins; Tannins; Terpanoids; Triterpenoids etc.	Antibacterial; Anti-inflammatory; Anti-oxidant; Anti-toxic etc.	Suma & Urooj, 2012; Dasgupta et al., 2016.
Solanum melongena L.	Argenine; Aspartic acid; Histidine; Nasunin; Protocatechuric acid; Solasodine; Solanoflavone etc.	Analgesic; Anti-asthmatic; Anti-pyretic; Anti-inflammatory; Anti-oxidant; Hypolipidemic; Hypotensive etc.	Mutalik et al., 2003; Das & Barua, 2013.
Solanum tuberosum L.	Amino Acids; Anthocyanins; Flavanols; Glycoalkaloids; Hydroxicinnamic acids; Phenolic acids etc.	Anti-anaemic; Anti-cardiovascular; Anti-inflammatory; Dermatological activities; Detoxifier etc.	Swee et al., 2013; Anjum Sahair et al., 2018.
Spinacia oleracea L.	Glycosides; Flavaonoids; Quinones; Phytosterols; Protiens; Terpenoids etc.	Anti-allergic; Anti-cardiovascular; Anti-inflammatory; Anti-microbial; Anti-cancer; Purgative activity.	Mane et al., 2015.
Syzygium cumini (L.) Skeels	Alkaloids; Jambosine; Phenolic acid; Steroids; Tannins; Terpenoids etc.	Anti-cancer; Anti-diabetic; Anti-diarrheal, Anti-inflammatory; Anti-microbial (bacterial, fungal & viral); Anti-pyretic; Anti-ulcer; Astringent; Cardio protective; Gastro protective; Hepatoprotective etc.	Bijauliya et al., 2018; Singh and Navneet, 2018.

Trachyspermum ammi (L.) Spr.	Carvacrol; p-cymene; γ-terpinine; Thymol etc.	Analgesic; Anthelmintic; Anti-hypersensitive; Anti-inflammatory; Anti-microbial (bacterial & fungal); Anti-oxidant; Anti-spasmodic; Diuretic; Hypotensive; Hepatoprotective; Insecticidal; Nematocidal etc.	Bairwa et al., 2012; Chahal et al., 2017.
Trichosanthes anguina L.	Cucurbitacins; Flavonoids; Glucosides; Isoflavone; Triterpenes; Oxalates; Phenols; Phylates etc.	Analgesic; Antihelmintic; Anti-arthritic; Anti-diabetic; Anti-bacterial; Anti-fertility, Anti-inflammatory; Anti-oxidant; Anti-spasmodic; Anti-ulcer; Cytotoxic; Gastroprotective; Hypoglycaemic; Hepatoprotective, Larvicidal activity etc.	Devi, 2017
Trigonella foenum–graecum L. (leaves)	Galactomannans; Lysine & Tryptophan protein; Vitamins A, B1, C & Nicotinic acid etc.	Anti-cancer; Anti-diabetic; Anti-inflammatory; Anti-microbial (bacterial & fungal); Anti-oxidant; Anti-ulcer; Aphrodisiac; Astringent; Carminative; Diuretic; Expectorant; Hepatoprotective; Hypoglycemic; Tonic; Vermifugal etc.	Moradi kor et al., 2013.
Triticum aestivum L.	Alkanoids; Glycosides; Flavonoids; Phenols; Saponins; Steroids; Tanins; Terpenoids etc.	Analgesic; Anti-hypersensitive; Appendicitis; Constipation; Colon diseases (Diverticulum); Cough depressant; Diabetes; Heart disease; Hyper-glycemic; Ischaemic; Obesity etc.	Kumar et al., 2011.
Urtica ardens Link.	Biflavanoids; Cartenoids; Coumarins; Fatty acids; Flavones; Isolectins; Lignans; Phenolic acids; Sterols; Scopoletins; Tanins; Terpenoids; etc.	Anti-inflammatory; Anti-microbial; Cardiovascular activity; Immune-booster; Neuromodulation, etc.	Rajput et al., 2018.
Vigna mungo (L.) Hepper	Alkaloids; Carbohydrates; Flavonoids; Saponins; Steroids; Tannins; Vitamin C	Anti-convulsant; Anti-inflammatory; Anti-oxidant; Analgesic; Immuno-stimulatory; Hypoglycemic, Hepatoprotective; Narcotic activity; Ulcerogenic.	Varma et al., 2013.
Vigna umbellata (Thunb.) Ohwi & Ohashi	Aglycone; Alkaloids; Chlorogenic acids; Flavonoids; Glycosides; Polyphenols; Saponins; Tannins; Triterpenoids; Vignalin etc.	Anti-helmenthic; Anti-bacterial; Anti-microbial; Anti-nociceptive; Hypocholesterlemic; Thrombolytic; anti-sickling etc.	Ibrahim et al., 2017.
Vigna unguiculata (L.) Walp	Amino acids; Anti-nutritional components (Haemagglutinin & Protease inhibitor); Fibers; Globulin; Protein; Pentosan; Phytosterol; Water soluble gum.	Antimicrobial (bacterial & fungal)l; Anti-oxidant; Anti-diabetes; Anti-hyperglycemic.	Gupta et al., 2016.

Zea mays L.	Niacin; Pyridoxine; Pantothenic acids; Riboflavin; Thiamine; Tryptamine etc.	Anti-carcinogenic; Anti-depressant; Anti-fatigue; Anti-microbial; Anti-neoplastic; Anti-obesitic; Anti-oxidant; Anti-tumour; Diuretic etc.	Ghete et al., 2019.
Zingiber officinale Roscoe	Volatile Compounds-sesquiterpene hydrocarbons (Zingeberene, Curcumene, Farnesene); Non-Volatile (Gingersol, Shogaols, Paradol) Proteoltic enzyme (Zingibain); Oleoresins; Vitamin B,C,E etc.	Anti-allergic; Anti-cancer (Breast cancer); Anti-carcinogenic; Anti-Inflammatory; Anti-malarial; Anti-microbial; Anti-oxidative; Cough suppressant; Gastrointestinal relief; Growth promoter; Hypoglycemic; Immuno-stimulant; Larvicidal etc.	Pour et al., 2014; Gupta and Sharma, 2014.
